# Reconstruction of insect hormone pathways in an aquatic firefly, *Sclerotia aquatilis* (Coleoptera: Lampyridae), using RNA-seq

**DOI:** 10.7717/peerj.7428

**Published:** 2019-08-02

**Authors:** Pornchanan Chanchay, Wanwipa Vongsangnak, Anchana Thancharoen, Ajaraporn Sriboonlert

**Affiliations:** 1 Department of Genetics, Faculty of Science, Kasetsart University, Bangkok, Thailand; 2 Department of Zoology, Faculty of Science, Kasetsart University, Bangkok, Thailand; 3 Omics Center for Agriculture, Bioresources, Food, and Health, Faculty of Science, Kasetsart University, Bangkok, Thailand; 4 Department of Entomology, Faculty of Agriculture, Kasetsart University, Bangkok, Thailand

**Keywords:** Ecdysteroid, Juvenile hormone, Transcriptome

## Abstract

Insect hormones: ecdysteroids and juvenile hormones have crucial functions during the regulation of different developmental pathways in insects. Insect metamorphosis is one of the primary pathways regulated by these hormones. The insect hormone biosynthetic pathway is conserved among arthropods, including insects, with some variations in the form of hormones used among each group of insects. In this study, the candidate genes involved in the insect hormone pathways and their functional roles were assessed in an aquatic firefly, *Sclerotia aquatilis* using a high-throughput RNA sequencing technique. Illumina next-generation sequencing (NGS) was used to generate transcriptome data for the different developmental stages (i.e., larva, pupa, and adult) of* S. aquatilis*. A total of 82,022 unigenes were generated across all different developmental stages. Functional annotation was performed for each gene, based on multiple biological databases, generating 46,230 unigenes. These unigenes were subsequently mapped using KEGG pathways. Accordingly, 221 protein-encoding genes involved in the insect hormone pathways were identified, including, *JHAMT, CYP15A1, JHE*, and Halloween family genes. Twenty potential gene candidates associated with the biosynthetic and degradation pathways for insect hormones were subjected to real-time PCR, reverse transcriptase PCR (RT-PCR) and sequencing analyses. The real-time PCR results showed similar expression patterns as those observed for transcriptome expression profiles for most of the examined genes. RT-PCR and Sanger sequencing confirmed the expressed coding sequences of these gene candidates. This study is the first to examine firefly insect hormone pathways, facilitating a better understanding of firefly growth and development.

## Introduction

Insects possess a rigid exoskeleton, which provides adaptability in the changing natural environment and has significantly contributed to insects’ evolutionary success (Cheong et al., 2015; Noh et al., 2016). Due to this rigid exoskeleton, insects require periodic molting in order to increase their sizes (Qu et al., 2018). Insect metamorphosis can be classified into either complete (holometabolous) or simple (hemimetabolous) metamorphosis. Steroid hormones are the crucial keys for controlling insect metamorphosis (Niwa & Niwa, 2016). These hormones regulate the expression of genes that are involved in the developmental transitions and reproduction (Jindra, Palli & Riddiford, 2013; Niwa & Niwa, 2016; Song et al., 2017). The identified hormones involved in insect metamorphosis are ecdysteroids (Niwa & Niwa, 2016) and sesquiterpenoids (Qu et al., 2018). Ecdysteroids are produced in the prothoracic gland and released into the haemolymph (Nijhout & Callier, 2015; Niwa & Niwa, 2016; Lin, Yu & Smagghe, 2018), and they are required for the molting process in arthropods (Song et al., 2017). In contrast, the sesquiterpenoid hormones, juvenile hormone (JH) and methyl farnesoate (MF), are two of the most crucial hormones for metamorphosis and are produced in the endocrine glands, the corpora allata (Cheong et al., 2015; Qu et al., 2018). Significant reductions in JH and MF hormone titers promote metamorphosis from larvae to adults, either via a pupal phase or directly through to an adult phase in the holometabolous and hemimetabolous metamorphosis, respectively (Qu et al., 2018). Not only are the JH and MF hormones crucial for insect metamorphosis, but they are also required for other developmental and reproductive processes, such as growth rate, body size, oogenesis, and gonad and ovarian maturation (Jindra, Palli & Riddiford, 2013; Song et al., 2017; Qu et al., 2018). JH has also been reported to be involved in longevity and insect behaviour (Trumbo, 2018; Qu et al., 2018). Because the specific levels of these hormones are crucial during each developmental stage, they must be tightly controlled using various factors (e.g., neuropeptides (Cheong et al., 2015) and microRNAs (miRNAs) (Qu et al., 2018)).

Insect developmental pathways, such as metamorphosis, growth, and reproduction, have been intensively studied for decades. Recently, rapid progress has been made due to the advancement of new sequencing technologies. However, the molecular mechanisms underlying insect development, particularly in Lampyridae, are still not fully understood. The primary focus of most research conducted for this group of insects has been the elucidation of their bioluminescence mechanisms (Viviani, Carmargo & Amaral, 2013; Vongsangnak, Chumnanpuen & Sriboonlert, 2016; Amaral, Silva & Viviani, 2017). However, due to the large amounts of data generated using NGS techniques, putative genes involved in other biological processes have simultaneously been identified in fireflies. In addition to putative genes associated with bioluminescence pathways, Amaral and colleagues (2017) reported that they identified gene products that were likely to be related to developmental processes in their studies of the Brazilian firefly, *Aspisoma lineatum.* Although, some information regarding the firefly developmental pathways has been generated, these data still require further analyses. Therefore, in this study, we aimed to generate transcriptome data from the three different developmental stages (i.e., larva, pupa, and adult) of an aquatic firefly, *S. aquatilis* to investigate the biosynthetic pathways for insect hormones of fireflies. Here, Illumina NGS was used to generate transcriptome data from the different developmental stages of *S. aquatilis,* and the data were further validated using quantitative real-time PCR. This study is the first report on the biosynthetic pathways for insect hormone in fireflies, facilitating a better understanding of firefly growth and development. This study provides valuable transcriptome data for the different developmental stages of *S. aquatilis* which can be used in future applications for both conservation and research purposes.

## Materials & Methods

### RNA extraction and sequencing

All *Sclerotia aquatilis* specimens used in this study were provided by Anchana Thancharoen (firefly rearing laboratory, Department of Entomology, Faculty of Agriculture, Kasetsart University). Total RNA extractions were performed according to Vongsangnak, Chumnanpuen & Sriboonlert (2016). The RNA extractions were prepared from six specimens for each developmental stage: larva (six specimens of 5th instar), pupa (six specimens), and adult (four female specimens and two male specimens). Two samples were pooled to make three replicates for cDNA library constructions. Quality and quantity of RNA samples were measured using an Agilent 2100 Bioanalyzer (Agilent Technologies, Santa Clara, CA, USA). Preparation of cDNA library and sequencing were performed according to Vongsangnak, Chumnanpuen & Sriboonlert (2016). RNA sequencing was performed using an Illumina Hiseq2000 Sequencing System (Illumina, San Diego, CA, USA) to generate paired-end reads with 100 base pairs (bp) read length.

### *De novo* transcriptome assembly

Low quality reads (i.e., reads with more than 20% of the base qualities being lower than 10, reads with adaptors and reads with unknown bases [N bases greater than than 5%]) were removed with FASTQC and Trimmomatic. Clean reads were assembled into Unigenes using Trinity v.2.0.6. (https://github.com/trinityrnaseq/trinityrnaseq), with parameter setting: –min_contig_length 150 (minimum 150 base of assembled contig length to report) –CPU 8 (8 CPUs to use) –min_kmer_cov 3 (minimum 3 count for K-mers to be assembled by Inchworm) –min_glue 3 (minimum 3 reads needed to glue two inchworm contigs together) –bfly_opts ’-V 5 (additional parameters to pass through to butterfly) –edget-hr=0.1 (set 0.1 as value to butterfly command) –stderr’ (standard error command to butterfly). Gene family clustering was subsequently performed using Tgicl clustering software v2.0.6.

### Functional annotation of transcripts

Functional annotation was performed against NCBI non-redundant protein sequences (NR; ftp://ftp.ncbi.nlm.nih.gov/blast/db), NCBI non-redundant nucleotide sequences (NT; ftp://ftp.ncbi.nlm.nih.gov/blast/db), clusters of orthologous groups of proteins (KOG/COG; ftp://ftp.ncbi.nih.gov/pub/COG/KOG), Swiss-Prot (http://ftp.ebi.ac.uk/pub/databases/swissprot) and Kyoto Encyclopedia of Genes and Genomes (KEGG; http://www.genome.jp/kegg), Gene Ontology (GO; http://geneontology.org) and IntroPro (v5.11–51.0; http://www.ebi.ac.uk/interpro/interproscan.html) databases using Blastn, Blastx (v.2.2.23; http://blast.ncbi.nlm.nih.gov/Blast.cgi), Diamond, Blast2GO (v2.5.0; https://www.blast2go.com) and InterPro Scan5 (v5.11–51.0; https://code.google.com/p/interproscan/wiki/Introduction). The results were filtered using an *E*-value of 1E-05 and a sequence identity of greater than 10%, and the best hit for each transcript was retained. Transdecoder (v.3.0.1; https://transdecoder.github.io) was used to identify the candidate coding area. The longest open reading frame (ORF) was extracted, and then the Pfam protein homologous sequences were searched by using the BLAST tool (v.2.2.23; http://blast.ncbi.nlm.nih.gov/Blast.cgi) in the SwissProt database (http://ftp.ebi.ac.uk/pub/databases/swissprot) and Hmmscan to predict the coding region. These programs were run using default parameter settings. All annotated transcripts were used to develop a Venn diagram, using jvenn (http://jvenn.toulouse.inra.fr/app/example.html) to represent the number of annotated transcripts expressed during the three stages of *S. aquatilis* development and other analyses.

### Comparative analysis of Unigenes among firefly species

The annotated transcripts which were each assigned a SwissProt ID based on the Swiss-Prot database and were used to compare the transcripts against the SwissProt IDs from three fireflies species, *Asymmetricata circumdata, Aquatica ficta,* and *Pyrocoelia pectoralis* (Wang et al., 2017), to identify orthologous genes among fireflies species.

### Identification of genes involved in insect hormone pathways

The amino acid sequences translated by Transdecoder were mapped to the KEGG database using Ghost KEGG Orthology and Links Annotation (KOALA) (https://www.kegg.jp/ghostkoala/). Those sequences that were mapped to the biosynthetic pathways for insect hormones were collected for further analysis. The expression profiles for these genes were visualised as a Heatmap, generated based on fragments per kilobase of transcript per million mapped reads (FPKM) values, using Morpheus, (https://software.broadinstitute.org/morpheus/) with a Pearson correlation hierarchical clustering, average linkage method. The significant differences in the FPKM values among the three stages were examined using a one-way analysis of variance (ANOVA) statistics and a post hoc test (Scheffe method) under the 95% confidence level, using SPSS software (IBM SPSS Statistics for Windows, Version 22.0; IBM Corp., Armonk, NY, USA).

These candidate genes were further analyzed to identify the most probable transcripts for each enzyme in the pathway. The candidate genes were BLASTed against the amino acid sequences of other insect species, i.e., *T. castaneum* (KEGG genes ID: tca:661876, tca:661964, tca:662004, tca:100142466, tca:656859, tca:657029, tca:657192, tca:657270, tca:657342, tca:657419, tca:657969, tca:658287, tca:658434, tca:658587, tca:658671, tca:658696, tca:659209, tca:659494, tca:103312192, tca:103314817, tca:656328, tca:659438, tca:661224, tca:661279, tca:100141982, tca:100142187, tca:662961, tca:658858, tca:658208, tca:100313955, tca:659305, tca:659375, tca:659506, tca:659572, tca:658081, tca:663127, tca:656884, tca:663098, tca:658665, tca:661451, tca:656794), *D. melanogaster* (sequence ID: CG15739, AF484413, AY079170, AF484414, U44753, NP_651725; Nyati et al., 2013; Warren et al., 2004; Warren et al., 2002; Petryk et al., 2003; Bassett et al., 1997), *A. aegypti* (sequence ID: GQ344797.1, GQ344798.1, GQ344799.1, KC243497, KC243498, KC243495, KC243496, KC243499; Mayoral et al., 2009; Rivera-Perez et al., 2013)*, B. mori* (sequence ID: AB124839, AB113578, AY377854, AY363308, AF287267, ABB58822, AB198340; Daimon et al., 2012; Shinoda & Itoyama, 2003; Zhang et al., 2005; Li et al., 2005; Hirai et al., 2002; Rewitz, O’Connor & Gilbert, 2007; Niwa et al., 2005)*, D. punctata* (sequence ID: AY509244 and AY509245; Helvig et al., 2004)*, C. fumiferana* (sequence ID: AF153367; Hirai et al., 2002) and *H. virescens* (sequence ID: AF037196; Hirai et al., 2002)*.* The best possible candidates for each enzyme-coding gene were selected based on the following criteria: BLASTP identity value greater than 20%; 1E-50 *E*-value cut off; FPKM values that were significantly different among the three developmental stages (tested by one-way ANOVA with *p* < 0.05); and positive detection in three other firefly species *A. circumdata, A. ficta*, and *P. pectoralis* (Wang et al., 2017). The identified candidates were then subjected to real-time PCR, RT-PCR, and sequencing.

### Validation of candidate genes expression using real-time PCR

The expression profiles of the selected candidate genes were examined using real-time PCR. Three individual specimens were used for each developmental stage (three fifth instar larvae of unknown sex; three pupae of unknown sex; three adults (two females and one male). Each specimen was pulverized into a powder with liquid nitrogen. Total RNA was extracted using TRIzol reagent (Invitrogen, Waltham, MA, USA), according to manufacturer’s protocol. The total RNA was subsequently treated with DNase I (Qiagen, Hilden, Germany) as per manufacturer’s protocol. Total RNA was converted into cDNA using RevertAid first strand cDNA kit (Thermo Scientific, Waltham, MA, USA). The reverse transcription reaction included 2 µg of total RNA, 10mM dNTP, 5x reaction buffer, RiboLock RNase Inhibitor, and RevertAid M-MuLV RT as described in manufacturer’s protocol and the reaction was incubated for 60 min at 42 °C and terminated at 70 °C for 5 min. Quantitative real-time PCR was performed using an Eppendorf Mastercycler ep realplex4 S real-time RT-PCR instrument (Eppendorf, Hauppauge, NY, USA) with Maxima SYBR green qPCR master mix (Thermo Scientific, Waltham, MA, USA). The quantitative real-time PCR reaction included cDNA template 0.5 µL, 0.3 µM of forward/reverse primers (File S1), Maxima SYBR green qPCR Master Mix (2x) no ROX 5 µL and water was added up to 10 µL. *Actin* (CL5997) was amplified as an internal control. The PCR reaction was initial denaturation for 10 min at 95 °C, followed by denaturation for 15 s at 95 °C, annealing for 30 s at 59 °C, and the last extension for 30 s at 72 °C. Three replicates were performed for each sample. The CT value from Real-time PCR was used to calculate a relative expression using the Δ ΔCT method. Significant differences of relative expression value among stage were tested with one-way ANOVA statistics and a post hoc test (Scheffe method) at *p*-value <0.05.

### Validation of candidate genes expression using RT-PCR and sequencing

The full-length sequences of the candidate genes were examined using RT-PCR, followed by sequencing. Complementary DNA obtained from the previously described real-time PCR experiment was used as the template during RT-PCR analysis. RT-PCR primers (File S1) were designed to amplify the longest ORFs in each candidate gene. PCR was performed using KOD-PLUS-NEO DNA polymerase (TOYOBO, Japan), according to manufacturer’s protocol. The PCR was performed using a Bio-rad T100™ Thermal Cycler (Bio-Rad, Hercules, CA, USA), with the following conditions: initial denaturation for 2 min at 94 °C, followed by 30 cycles of denaturation for 10 s at 98 °C, annealing for 30 s at 50 °C, and extension for 1 min at 68 °C. PCR products were subsequently sent for sequencing at Macrogen (South Korea).

## Results

### *De novo* transcriptome assembly

A total of 711.9 Mb of raw reads were obtained from the Illumina HiSeq 2000 sequencing of nine specimens across the three developmental stages of *S. aquatilis* (Table 1). After adapter trimming and the removal of contaminating sequences and low-quality sequences, a total of 592.32 Mb of clean reads were retained. These clean reads were assembled into 383,922 high-quality contigs. Unigenes were obtained by removing the transcript abundance. The GC contents for all samples ranged from 36–38%. A total of 82,022 unigenes were retained, with a total length, average length, N50, and GC content of 117,432,935 bps, 1,394 bps, 2,666 bps, and 35.65%, respectively. The clean reads from the nine specimens of *S. aquatilis* used in this study were deposited into the NCBI SRA database under accession number PRJNA525262.

**Table 1:** Overview of transcriptome data. Transcriptome data of three different developmental stages: larval, pupal, and adult of *S. aquatilis* were obtained from Illumina Hiseq 2000 platform.

Samples	Larva	Larva	Larva	Pupa	Pupa	Pupa	Adult	Adult	Adult
	01	02	03	01	02	03	01	02	03
Total Raw Reads(Mb)	81.64	78.38	78.38	80	76.75	80.01	78.38	78.38	80
Total Clean Reads(Mb)	65.6	65.78	65.76	65.97	65.39	65.91	65.63	66.16	66.12
Total Clean Bases(Gb)	6.56	6.58	6.58	6.6	6.54	6.54	6.56	6.62	6.61
Clean Reads Q20(%)	97.2	97.05	97.05	97.31	97.08	97.12	97.06	96.86	96.94
GC(%)	37.28	37	36.92	36.74	36.84	36.84	37.05	36.97	36.65
Total Transcripts	46591	47260	44584	49185	44050	52326	35280	33835	50608
Mean Length of Transcripts	942	1036	987	1007	1050	971	1049	1082	1064
N50 of Transcripts	1982	2118	1974	2137	2165	2077	2071	2144	2308
Total Unigenes	34262	34721	33399	34949	32406	37057	26141	25041	36390
Mean Length of Unigenes	1108	1220	1148	1214	1247	1187	1254	1288	1277
N50 of Unigenes	2093	2218	2080	2270	2288	2239	2175	2240	2439

### Gene annotation

**Figure 1:**
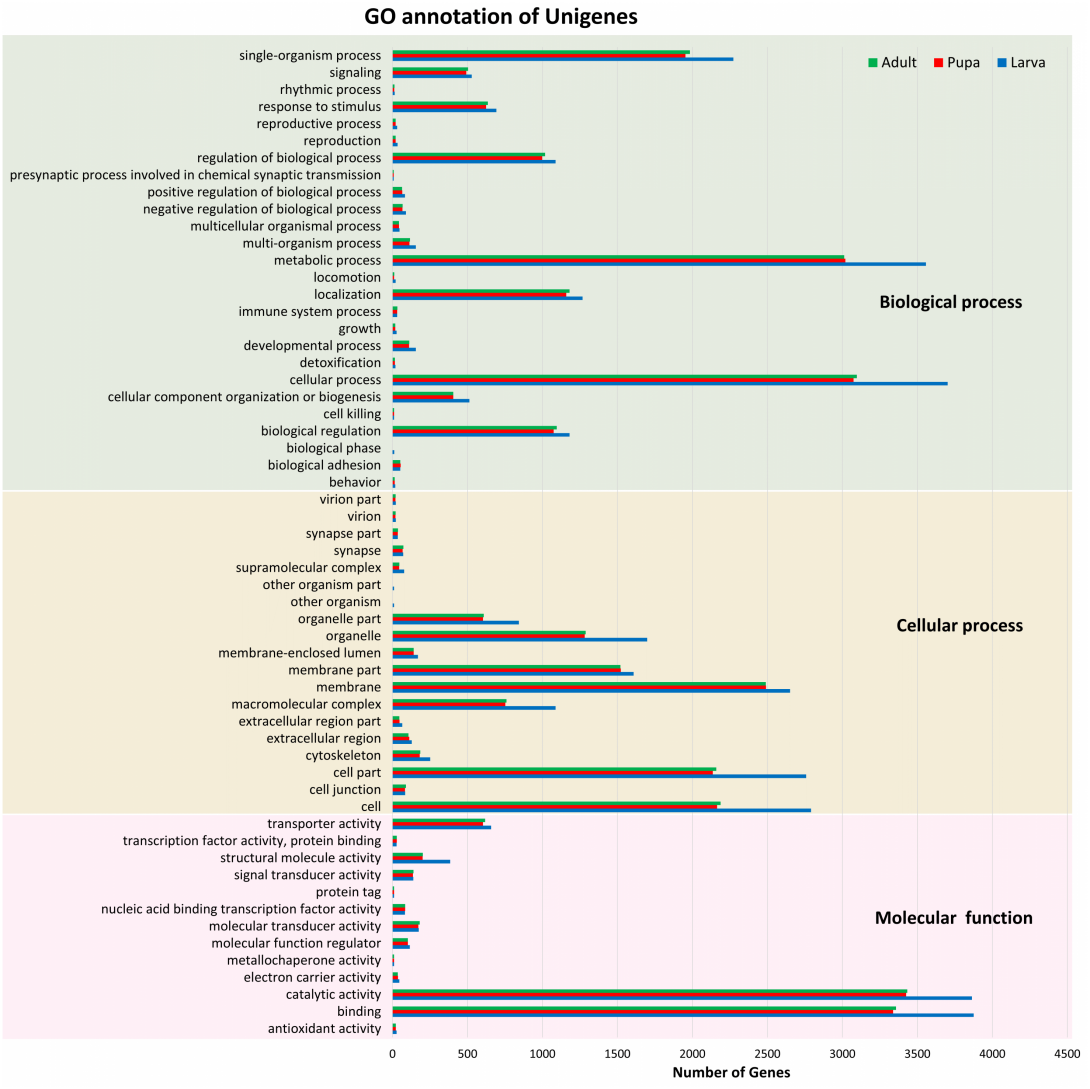
Gene Ontology (GO) annotation of unigenes. Functional annotation of unigenes obtained from three developmental stages (larva, blue; pupa, red; adult, green) of *S. aquatilis*. The unigenes were categorized into three main categories: biological process, cellular component, and molecular function, according to GO functional category.

Of these 82,022 assembled unigenes, 46,230 (56.36%) were annotated in the following seven databases: Nr (44,959), Nt (9,908), SwissProt (34,626), KEGG (35,393), KOG (33,407), InterPro (35,813) and GO (9,278) databases (File S2). These annotated transcripts were categorized into three primary functional groups: biological process, cellular component, and molecular function, according to GO annotations (Fig. 1). The functional category that contained the highest number of unigenes was the biological process (16,752). Within this biological process category, most of the unigenes were subcategorized into the metabolic (3,784) or cellular (3,932) processes. The results also showed that the number of unigenes among the three stages were distributed similarly across the various subcategories; for example, the number of unigenes in the metabolic process categories larval, pupal, and adult stages were 3,556, 3,019, and 3,012, respectively.

**Figure 2:**
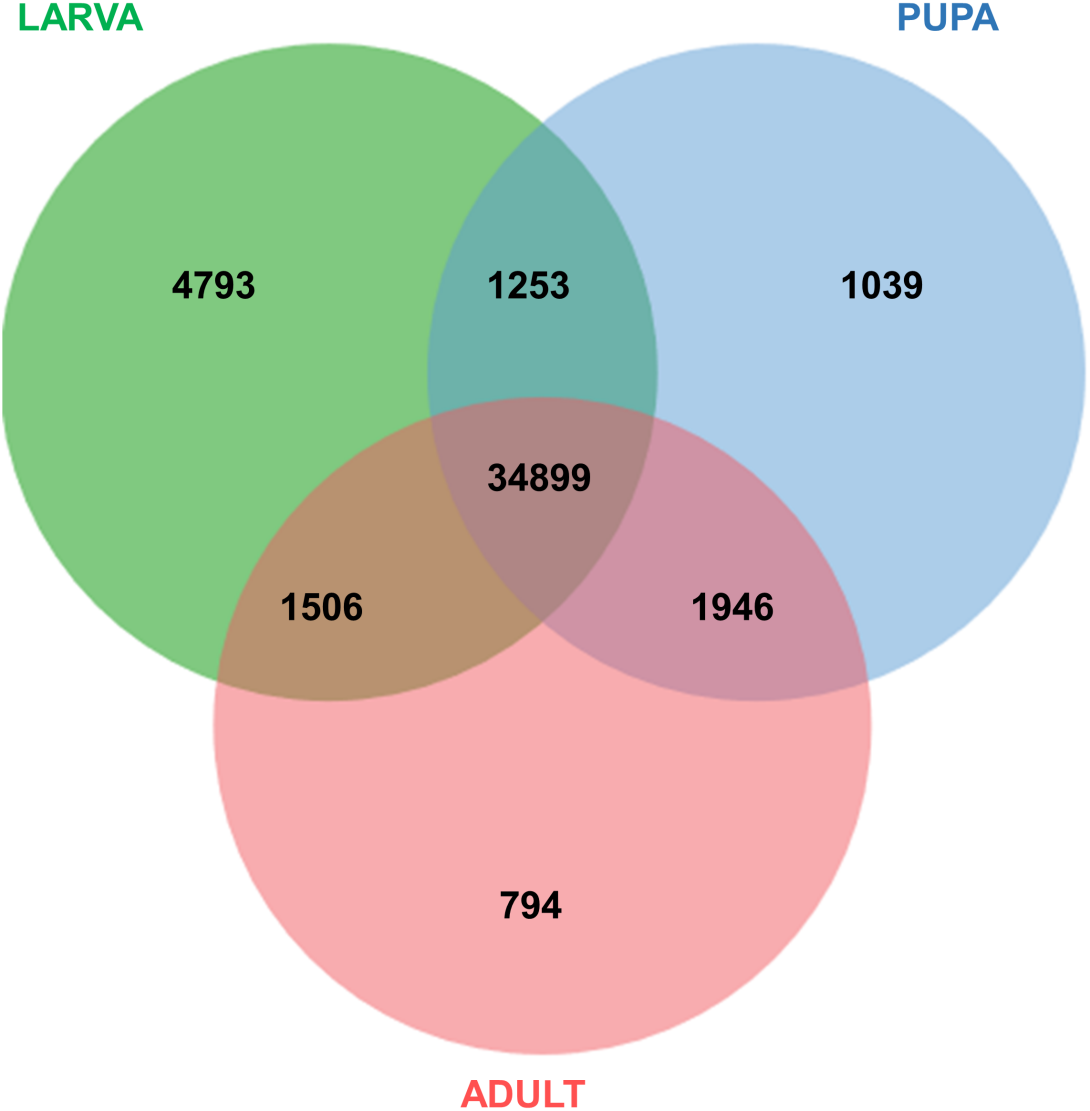
Venn diagram of annotated transcripts among the three stages of *S. aquatilis* development. Venn diagram illustrating the number of annotated transcripts expressed in larval, pupal, and adult stages of *S. aquatilis*.

**Figure 3:**
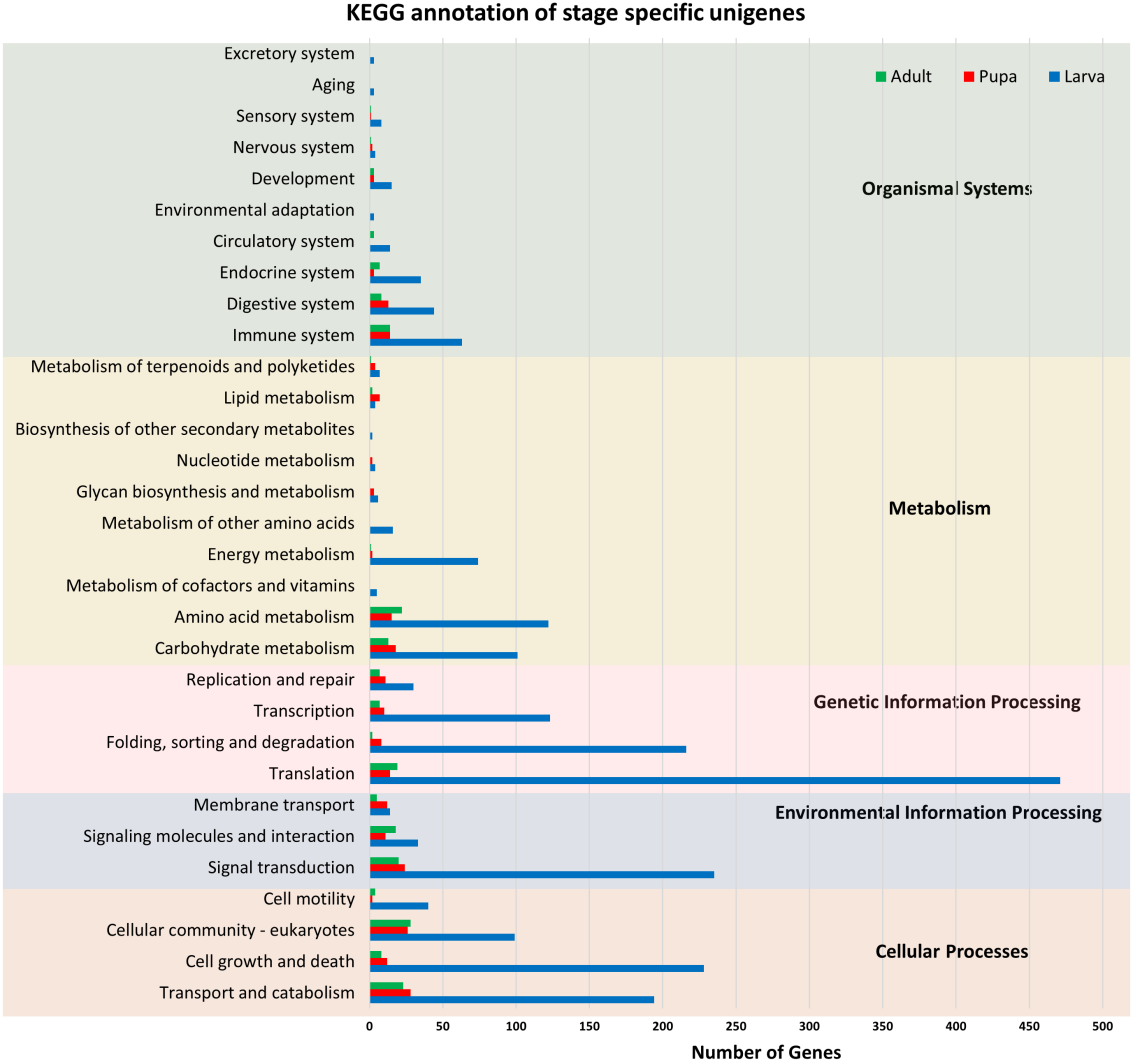
KEGG functional annotation of specifically expressed unigenes of each developmental stage of *S. aquatilis*. Stage-specific unigenes (adult, green; pupa, red; larva, blue) were categorized into five functional categories based on KEGG: cellular processes, environmental information processing, genetic information processing, metabolism, and organismal systems.

All of the annotated transcripts obtained from all of the databases were then combined and compared among the three different developmental stages of *S. aquatilis*. A total of 34,899 unigenes were found to be commonly expressed across three different developmental stages. In contrast, 4,793, 1,039, and 794 unigenes were specifically expressed during the larval, pupal, and adult stages, respectively (Fig. 2). The larval and pupal stages shared 1,253 unigenes, the pupal and adult stages shared 1,946 unigenes and the larval and adult stage shared 1,506 unigenes. These specifically expressed unigenes were then catagorized into functional catagories, based on the KEGG database (Fig. 3). During the larval stage, the highest numbers of specifically expressed unigenes were identified in the genetic information processing category (840 unigenes) and the translation subcategory (471 unigenes). During the pupal stage, the highest number of specific unigenes was identified in the metabolism category (91 unigenes). During the adult stage, the highest numbers of specific unigenes were identified in the cellular processes (63 unigenes) and the cellular community subcategory (28 unigenes). The number of specifically expressed unigenes associated with lipid metabolism (seven unigenes) was relatively high during the pupal stage compared with the larval and adult stages.

### Comparative analysis of Unigenes among firefly species

**Figure 4:**
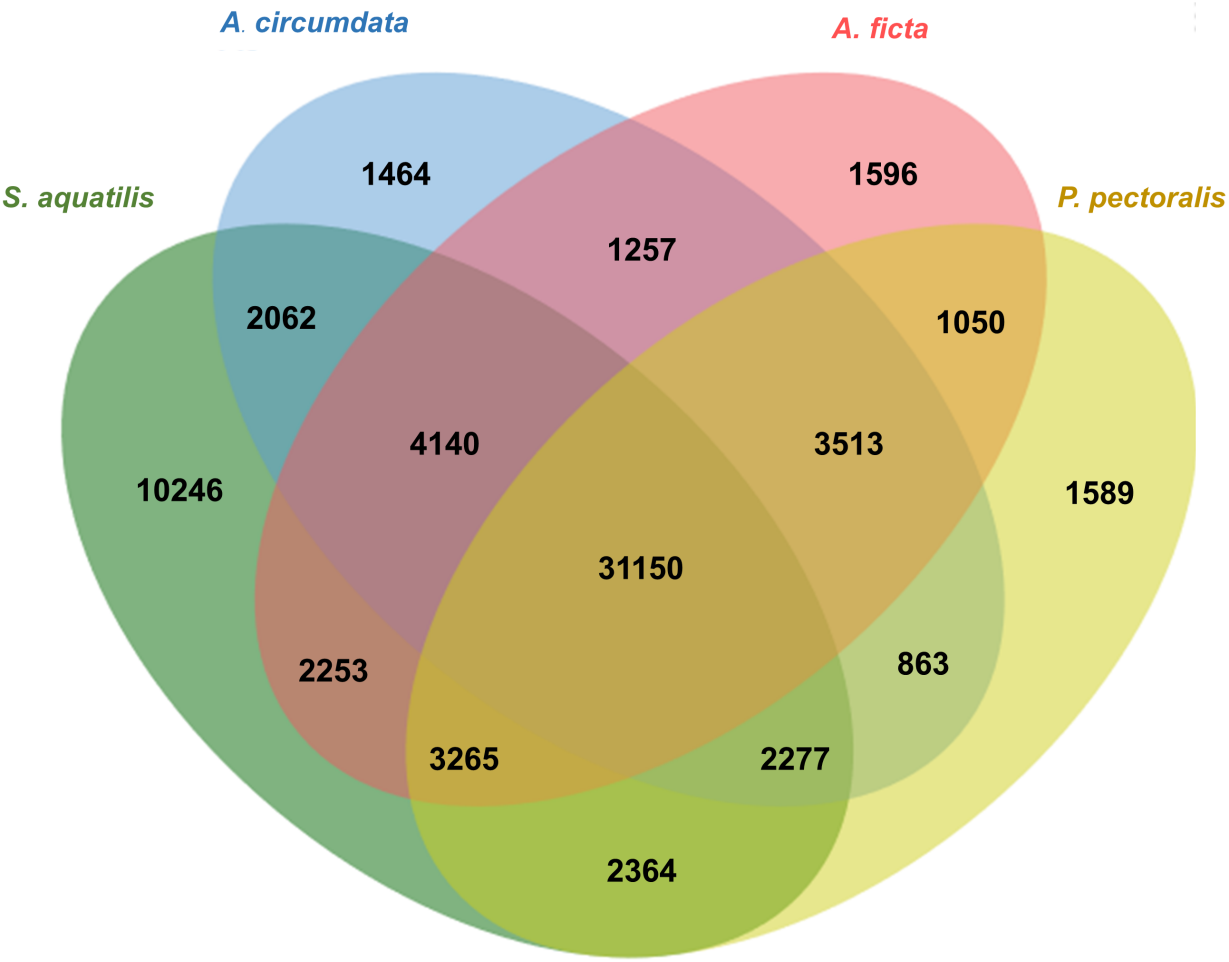
Venn diagram of annotated unigenes among four firefly species. Venn diagram illustrating the number of annotated unigenes among four firefly species: *A. circumdata, A. ficta, P. pectoralis* (Wang et al., 2017) and *S. aquatilis* (this study).

Orthologous genes among four firefly species, *A. circumdata, A. ficta, P. pectoralis,* (Wang et al., 2017) and *S. aquatilis* (this study) were identified. The Swissprot IDs of the three firefly species, *A. circumdata, A. ficta,* and *P. pectoralis* were obtained from (Wang et al., 2017) and compared with *S. aquatilis* unigenes in the present study. The analysis showed 1,464, 1,596, 1,589, and 10,246 unigenes found specifically in *A. circumdata, A. ficta, P. pectoralis,* and *S. aquatilis,* respectively. A total of 31,150 unigenes were found in all four species (Fig. 4; File S3).

### Identification of genes involved in insect hormone pathways

**Figure 5:**
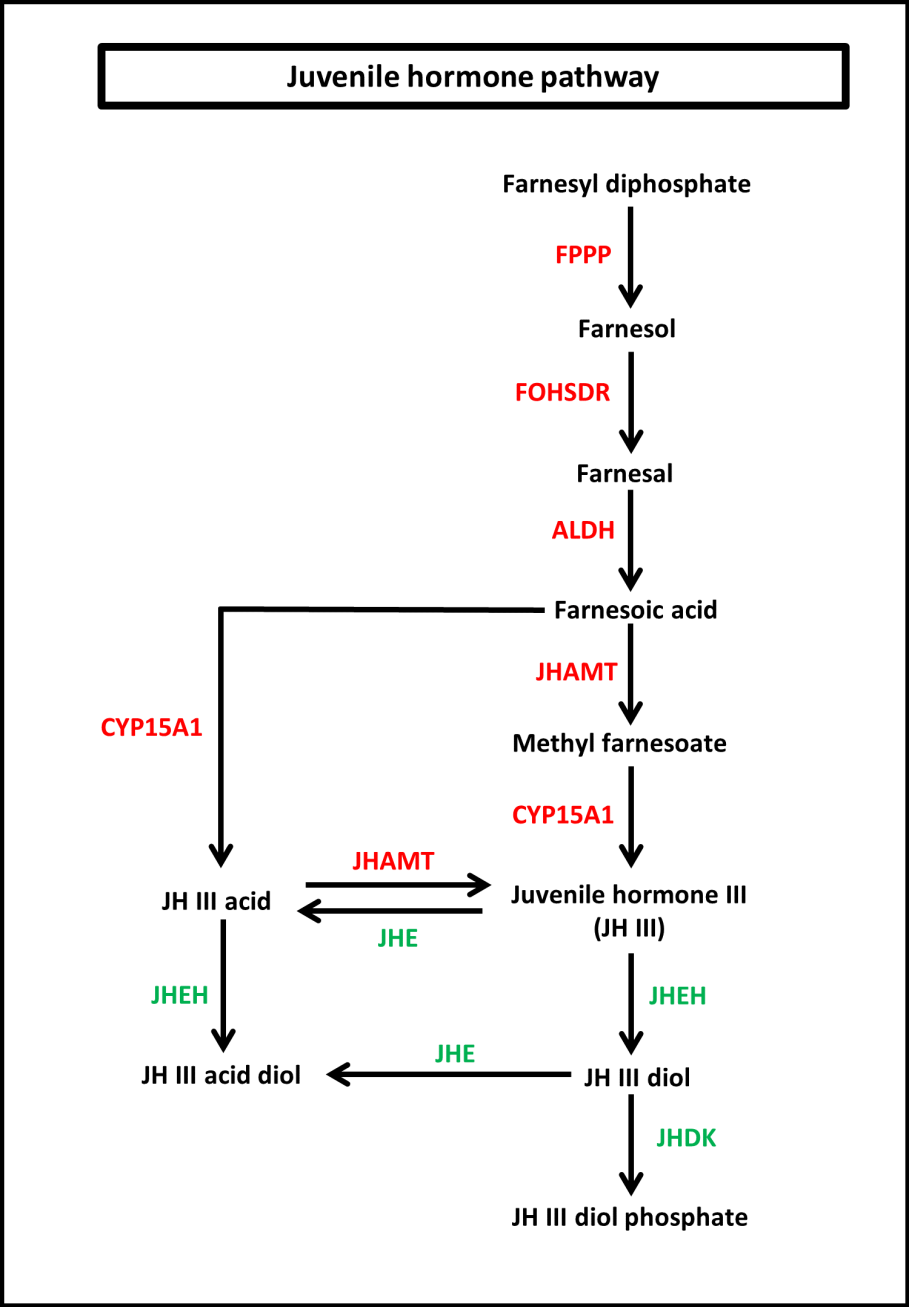
Juvenile hormone (JH) pathway of *S. aquatilis*. JH pathway of *S. aquatilis* was reconstructed by mapping the annotated proteins to KEGG pathway. Enzymes involved in *S. aquatilis* JH biosynthetic pathway are shown in red and enzymes involved in JH degradation are shown in green.

**Figure 6:**
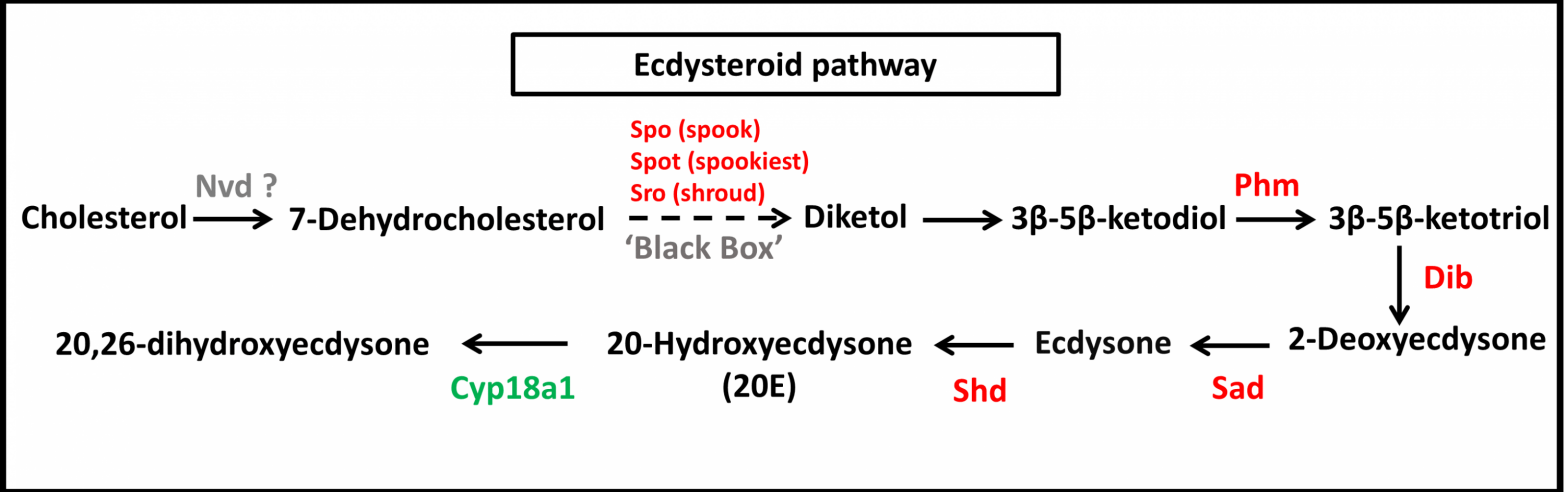
Molting hormone (ecdysteroid) pathway of *S. aquatilis*. Ecdysteroid pathway of *S. aquatilis* was reconstructed by mapping annotated proteins to KEGG pathway. Enzymes involved in *S. aquatilis* ecdysteroid biosynthetic pathway are shown in red and an enzyme involved in ecdysone degradation is shown in green.

To identify the protein coding genes involved in the pathways of insect hormones, all unigenes identified in *S. aquatilis* were mapped to the KEGG pathway database. A total of 221 transcripts were identified as being involved in the biosynthetic and degradation pathways of insect hormones (Table 2; File S2) with percent identities greater than 20 and *E*-value of 1E-05 set as the thresholds (Figs. 5 and 6). Of these 221 transcripts, 191 transcripts were identified in the JH biosynthetic and degradation pathways and 30 transcripts were identified in the ecdysteriod biosynthetic and degradation pathways. In the JH biosynthetic pathway, 130 transcripts were mapped to five enzymes, including farnesyl diphosphate phosphatase (FPPP; nine transcripts), NADP^+^-dependent farnesol dehydrogenase (FOHSDR; 56 transcripts), aldehyde dehydrogenase (ALDH; 23 transcripts), juvenile hormone-III synthase (JHAMT; 29 transcripts), and methyl farnesoate epoxidase/farnesoate epoxidase (CYP15A1; 13 transcripts). In JH degradation pathway, 61 transcripts were mapped to three enzymes comprising of juvenile-hormone esterase (JHE; 56 transcripts), juvenile hormone epoxide hydrolase (JHEH; three transcripts), and juvenile hormone diol kinase (JHDK; two transcripts). These enzymes were similar to the enzymes identified in the model coleopteran insect, *Tribolium,* except for the JHDK, which was not detected in the *Tribolium* but was found in *Bombyx mori* (Li et al., 2005) and *Manduca sexta* (Maxwell et al., 2002; Maxwell, Welch & Schooley, 2002). The differential expression of these 191 transcripts were also compared among the three developmental stages, larval, pupal and adult. FPKM values from the 191 transcripts were analyzed using one-way ANOVA statistics and a post hoc test (Scheffe method), at a confidence level of 95%. Only 34 transcripts were found to be significantly different.

**Table 2:** Annotated transcripts involved in the insect hormone pathways of fireflies. Transcripts from four firefly species, *S. aquatilis* (this study), *A. circumdata*, *A. ficta*, and *P. pactoralis* (Wang et al., 2017), were annotated as being involved in the insect hormone pathways.

KO ID	Protein name	Number of transcripts (based on KEGG)	Swiss-Prot ID	Number of transcripts (based on Swiss-Prot)			
		***S. aquatilis***		***S. aquatilis***	***A. circumdata***	***A. ficta***	***P. pectoralis***
K21013	FPPP	9	A6NDG6	5	0	0	0
			Q8CHP8	2	1	1	0
			Q5F4B1	1	0	0	0
			Q2T9S4	1	0	3	1
K15890	FOHSDR	56	Q3U0B3	23	10	8	0
			Q71R50	13	2	2	4
			Q3ZBV9	8	14	14	23
			Q6UWP2	11	3	4	3
K00128	ALDH	23	Q60HH8	3	0	1	0
			P43353	2	0	0	0
			P11884	2	1	1	0
			Q8IZ83	2	0	0	0
			Q2XQV4	2	0	0	0
			O94788	1	0	0	0
			P20000	1	0	0	0
			P51648	1	0	0	0
			P27463	1	0	0	1
K10718	JHAMT	29	Q9NBX5	1	18	9	1
			Q9TYP1	1	0	0	0
			Q54XD0	1	0	2	0
			Q91WU5	1	0	0	0
			A2APY7	1	1	0	0
K14937	CYP15A1/C1	13	Q9VW43	5	2	4	6
			P11509	1	0	0	0
			Q9V399	1	1	1	14
			Q9VG17	5	4	1	2
			Q6VVW9	1	0	0	0
K01063	JHE	56	B2D0J5	41	26	23	35
			Q91WG0	1	0	0	0
			P35502	9	10	8	17
			P35501	1	5	4	0
			Q92035	1	0	0	2
			P12992	1	0	0	0
			P81429	1	2	1	3
			P16303	1	0	0	0
K10719	JHEH	3	P07687	1	0	0	0
			Q8MZR5	1	1	2	0
			Q8MZR6	1	1	2	2
K15825	JHDK	2	Q10131	2	1	0	1
K14939	Spo, Spot	2	Q9VRM7	2	2	3	1
K10720	Phm	1	Q9VWR5	1	0	2	3
K10721	Dib	16	Q9NGX9	14	1	1	1
			Q9I7M2	1	1	1	1
			Q9V5L3	3	0	2	0
K10722	Sad, Cyp315a1	1	Q9VGH1	1	1	1	1
K10723	Shd, Cyp314a1	4	Q9VUF8	2	1	1	1
K14985	Cyp18a1	3	Q95078	3	1	1	1
K00019	Sro	3	Q62730	1	0	0	1
			Q7TQA3	2	2	1	1
			Q02338	1	1	1	1

In the ecdysteriod pathway, 30 transcripts were mapped to eight enzymes; cytochrome P450 family 307 subfamily A (Spook [Spo]; 1 transcript), cytochrome P450 family 307 subfamily B (Spookiest [Spot]; 1 transcript), short-chain dehydrogenase/reductase (Shroud [Sro]; 3 transcripts), ecdysteroid 25-hydroxylase (Phantom [Phm]; 1 transcript), ecdysteroid 22-hydroxylase (Disembodied [Dib]; 16 transcripts), ecdysteroid 2-hydroxylase (Shadow [Sad]; 1 transcript), ecdysone 20-monooxygenase (Shade [Shd]; 4 transcripts), and 26-hydroxylase (Cyp18a1; three transcripts) (Fig. 6). Almost all members of the insect ecdysterol Halloween gene family (*Spo, Spot, Phm, Dib, Sad,* and *Shd*) were identified in *S. aquatilis*, except for *Neverland* (*Nvd*). According to the KEGG pathway, this Nvd enzyme is also absent from the model coleopteran insect, *T. castaneum*. The expression profiles of these 30 transcripts were analyzed using their FPKM values. The significant differences among the differential expression profiles were analyzed using one-way ANOVA. However, the expression levels of all these transcripts were not significantly different among the three stages, except for *Sro* (Unigene21593).

Of these 191 transcripts identified in the JH pathway and the 30 transcripts in the ecdysone pathway, only twenty most probable candidates from the JH (eleven transcripts) and the ecdysone (nine transcripts) pathways were selected for further analyses. These twenty candidates were chosen based on at least one of the following criteria: (i) the protein coding gene transcript was detected in all four firefly species (*S. aquatilis* [this study], *A. circumdata, A. ficta* and *P. pectoralis* (Wang et al., 2017)); (ii) the gene was returned as a best hits when BLASTed against the *Tribolium* hormone enzymes; and (iii) the gene was differentially expressed among the three stages of development.

### Validation of differential gene expression by quantitative real-time PCR, RT-PCR, and sequencing

**Figure 7:**
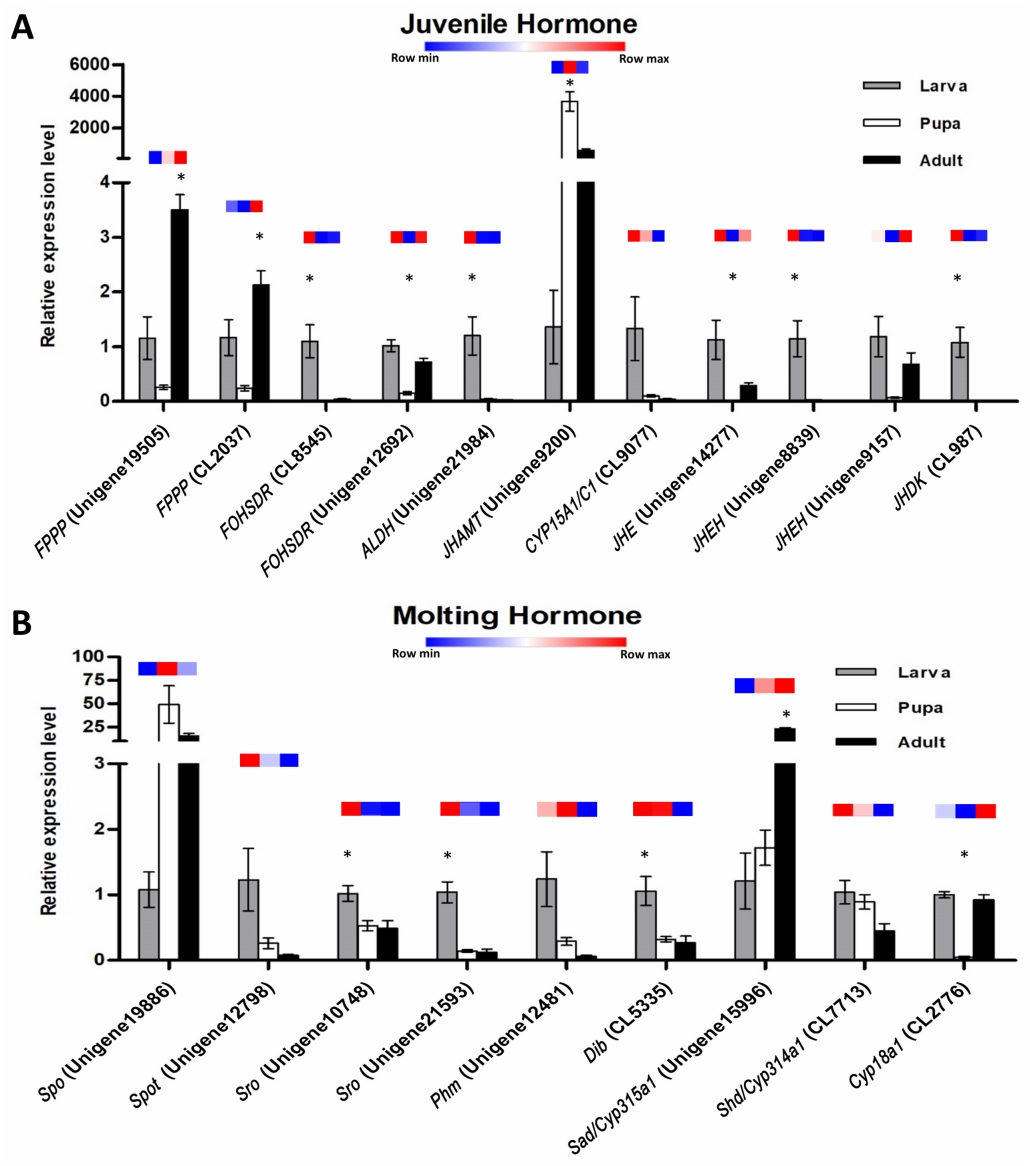
Quantitative real-time PCR analysis of candidate genes involved in *S. aquatilis* insect hormone pathways. (A) Real-time PCR analyses of eleven putative unigenes involved in the juvenile hormone pathway. (B) Real-time PCR analyses of nine putative unigenes involved in the ecdysteroid pathway. Color keys shown above each set of bar graphs demonstrate the expression profiles based on the FPKM value derived from the transcriptome analysis. The results are presented as the means ± SD. *Significant difference among the three developmental stages (*P* < 0.05).

**Figure 8:**
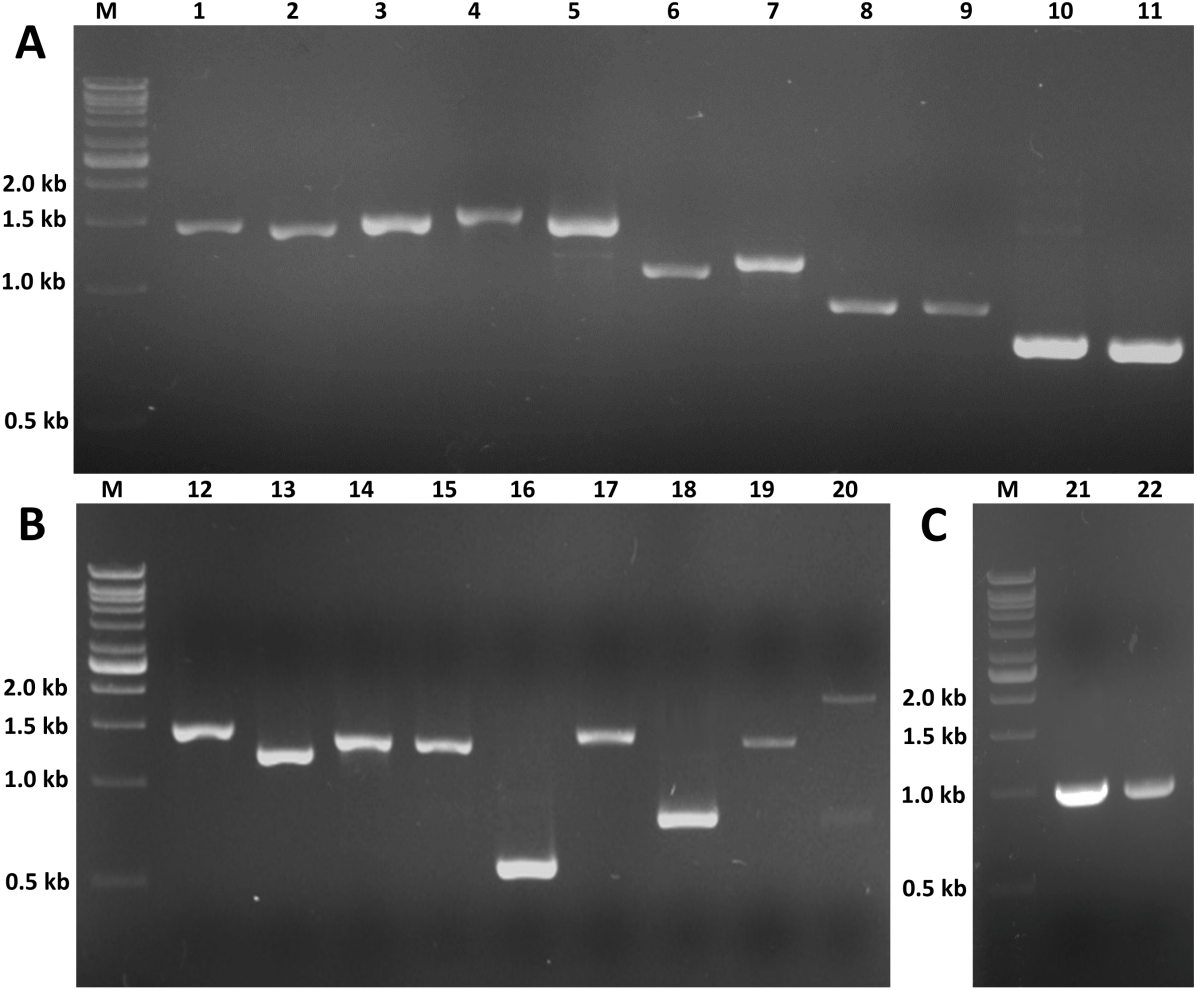
RT-PCR of candidate genes involved in insect hormone pathways. RT-PCR was performed to validate the expression of twenty selected candidate genes proposed to be involved in JH and ecdysone pathways. RT-PCR products of expected sizes were amplified except for lane 20 (Unigenen12798 [*Spot*]). (A) M: 1 kb DNA ladder; 1: Unigene12481 (*Phm*, 1,455 bp); 2: Unigene15996 (*Sad*, 1,422 bp); 3: CL7713 (*Shd*, 1,479 bp); 4: CL2776 (*Cyp18a1*, 1,587 bp); 5: CL5335 (*Dib*, 1,506 bp); 6: Unigene10748 (*Sro*, 1,110 bp); 7: Unigene12593 (*Sro*, 1,182 bp); 8: Unigene19505 (*FPPP*, 915 bp); 9: CL2037 (*FPPP*, 918 bp); 10: CL8545 (*FOHSDR*, 765 bp); 11: Unigene12692 (*FOHSDR*, 750 bp). (B) M: 1 kb DNA ladder; 12: CL9077 (*CYP15A1*, 1,521 bp); 13: Unigene14277 (*JHE*, 1,245 bp); 14: Unigene8839 (*JHEH*, 1,389 bp); 15: Unigene9157 (*JHEH*, 1,383 bp); 16: CL987 (*JHDK*, 549 bp); 17: Unigene21984 (*ALDH*, 1,494 bp); 18: Unigene9200 (*JHAMT*, 810 bp); 19: Unigene19886 (*Spo*, 1,455 bp); 20: Unigene12798 (*Spot*, 2,000 and 750 bp [expected size: 1,476 bp]). (C) M: 1 kb DNA ladder; 21: partial Unigene19886 (*Spo*, 1,038 bp); 22: partial Unigene12798 (*Spot*, 1,011 bp).

The expression profiles of these twenty potential candidates were validated using real-time PCR. *Actin* (CL5997) was used as a reference gene, due to its relatively stable expression in comparison with the other house keeping genes identified in this study. Real-time PCR results for most of the selected genes coincided with the expression data obtained from the transcriptome analysis (Fig. 7; File S4). However, the real-time PCR expression patterns for *FPPP* (Unigene19505), *JHEH* (Unigene9157), *Phm* (Unigene12481), and *Cyp18a1* (CL2776) among the three stages of *S. aquatilis* development slighlty deviated fom the expression patterns obtained from the transcriptome analysis. Moreover, RT-PCR and Sanger sequencing were also performed to analyze the full-length coding sequences of these transcripts. RT-PCR products of an expected sizes were successfully amplified for all of the selected candidates, except for Unigene12798 (*Spot*) (Figs. 8A and 8B). The nucleotide sequences for most of these candidates were nearly identical to the sequences obtained from the transcriptome analysis (File S5). However, the RT-PCR product for Unigene12798 (*Spot*) showed two bands of approximately 2,000 and 750 bp, instead of the expected 1,476 bp band. Sanger sequencing results for both Unigene12798 (*Spot*) and Unigene19886 (*Spo*) showed mixed peaks and were unable to be read. Thus, the primers for these two genes were redesigned and, subsequently, the partial coding sequences of *spo* (1,038bp) and *spot* (1,011bp) were able to be amplified and sequenced (Fig. 8C).

## Discussion

Fireflies are one of the most intriguing insects on earth. Their bioluminescence ability is not only mesmerizing but also beneficial and has been used for various applications (Gu et al., 2019; Tang et al., 2019; Wang et al., 2019). Although many studies have been conducted on this remarkable insect, the knowledge regarding the firefly developmental processes at the molecular level remain lacking. In this study, the primary pathway underlying firefly metamorphosis was elucidated. The transcriptome data for the three stages of firefly development were obtained from an aquatic firefly, *S. aquatilis*. This firefly species is commonly found in Thailand (Thancharoen et al., 2007) and has been successfully reared in the laboratory, facilitating specimen acquisition for this study. However, we were unable to obtain a sufficient numbers of eggs to perform RNA sequencing. Therefore, this stage of development was omitted from this study.

The raw read sequences obtained from the three stages of *S. aquatilis* were of a high-quality, and a total number of 82,022 unigenes were assembled. The number of unigenes obtained in this study was higher than the previously reported data for *S. aquatilis* (39,730; Vongsangnak, Chumnanpuen & Sriboonlert, 2016) and other firefly species, *A. circumdata* (24,275), *A. ficta* (31,520), and *P. pectoralis* (31,356; Wang et al., 2017), because different firefly developmental stages were included in this study, whereas only the larval stage was used in other studies; thus, more transcripts could be identified. Of these 82,022 unigenes, 46,230 unigenes (56.36%) were annotated, while totals of 15,352 (63.24%), 17,480 (55.46%), and 15,342 (48.93%) unigenes were annotated from *A. circumdata, A. ficta,* and *P. pectoralis*, respectively (Wang et al., 2017). In this study, we obtained a three-fold increased number of annotated unigenes than reported by previous studies, which should provide more information regarding the important biological processes required for firefly growth and development. The expression analyses among the three stages of development revealed a high number of transcripts in the larval stage compared with the pupal and adult stages, with 4,793 specifically expressed transcripts identified for the larval stage. The adult stage showed the lowest number of specifically expressed genes, with 794 transcripts. The high number of expressed genes in the larval stage may due to the larval stage being the dominant stage of development, taking the longest time and representing the most active stage of the life cycle (Thancharoen, 2007). During the pupal stage, the *S. aquatilis* is less active; none of the stage-specific genes associated with specific categories (e.g., detoxification, immune system process, behavior, locomotion, rhythmic process) were identified in the GO annotation result. The identified genes that were specifically expressed during the pupal stage were categorized into basic molecular and biological processes. During the adult stage, the primary activities are mating and reproduction. In most short-lived adult firefly species, including *S. aquatilis*, the adults no longer searching for prey, instead feeding only on liquid foods such as nectar and plant sap (Othman et al., 2018). However, some firefly species (e.g., the females of the genus *Photuris*) have methods for acquiring prey by consuming the adult males of another genus, *Photinus* to acquire a defensive compound (lucibufagin) in order to protect their eggs (Eisner et al., 1997; González, Hare & Eisner, 1999). Among these stage-specific transcripts, the genes involved in the insect hormone biosynthetic pathways have distinct expression patterns (Figs. 5 and 6). These stage-specific expression profiles observed for the ecdysteroid- and JH-related genes were also observed in the transcriptome study of the mealy bug, *Phenacoccus solenopsis* (Arya et al., 2018).

Insect metamorphosis is an important developmental process that affects the survival rates of many insects (Song et al., 2017). The two crucial groups of hormones that play the key roles during insect metamorphosis are juvenile (sequiterpenoids) and steroid hormones (ecdysteroids) (Cheong et al., 2015). These hormones are not only vital for metamorphosis but are also vital for growth, and reproduction (Jindra, Palli & Riddiford, 2013; Song et al., 2017). In this study, the JHs and the ecdysteroids biosynthetic and degradation pathways were revealed in firefly for the first time. In the JH pathway, farnesyl diphosphate is converted into farnesol, farnesal and farnesoic acid (FA) by FPPP, FOHSDR, and ALDH, respectively. FA is thought to be involved in reproduction in crustacians. FA is required for the production of vitellogenin during oocyte maturation (Bellés, Martín & Piulachs, 2005; Mak et al., 2005; Cheong et al., 2015). However, vitellogenin production in insects requires JH and its derivatives (Ismail et al., 1998). Different forms of JH have been identified in various insect taxa; JH-I, JH-II, and JH-0 (exclusive to Lepidoptera), JH-III skipped bisepoxide (Hemiptera) , JH-III bisepoxide (Diptera), and JH-III (most insects) (Bergot et al., 1980; Yin et al., 1995; Bellés, Martín & Piulachs, 2005; Kotaki et al., 2009; Cheong et al., 2015; Verlinden et al., 2015). In *S. aquatilis*, all of the enzymes required for the production of JH-III have been identified (Fig. 5). The FA is converted into JH, either via JH analog (JHA) by CYP15A1 and JHAMT or via methyl farnesoate (MF) by the same enzymes. In the lepidopterans, FA is converted to JH via JH-III acid (Daimon et al., 2012; Qu et al., 2018). In cockroaches and locusts, FA is converted to MF by JHAMT and then further converted to JH by CYP15A1 (Helvig et al., 2004; Marchal et al., 2011; Qu et al., 2018). In coleoptrans, both JHAMT and CYP15A1 have been identified in the coleopteran model, *Tribolium*, (Minakuchi et al., 2015) as well as in the firefly, as demonstrated by the current study. The transcriptional regulation of JHAMT was reported to be critical for *Tribolium* metamorphosis (Minakuchi et al., 2008); however, CYP15A1 RNAi-knockdown in *Tribolium* did not result in precocious metamorphosis (Minakuchi et al., 2015). *S. aquatilis* CYP15A1, in the present study, demonstrated a similar expression profile to that reported for *Tribolium* CYP15A1, with relatively high mRNA abundance during the final larval instar and constitutively expressed at a low level during the other developmental stages (Minakuchi et al., 2015). JHAMT is one of the key enzymes that plays a crucial role for the regulation of the JH titre. The function of JHAMT during metamarphosis has been demonstrated in various insect species. JHAMT is required for larval-pupal metamorphosis and body size control in *Tribolium*. In the Colorado potato beetle, *Leptinotarsa decemlineata*, the knockdown of JHAMT leads to higher death rates, weight loss in larvae, and precocious and impaired pupation, with reduced eclosion rates (Qu et al., 2018). In the present study, the expression levels of all enzymes associated with JH biosynthesis and degradation were detected during the larval stages of *S. aquatilis*, including JHDK. JHDK is the only enzyme associated with the JH pathway that has not been previously reported in the KEGG pathway for *T. castaneum*. However, JHDK enzyme was identified in another coleopteran insect, *L. decemlineata* (Colorado potato beetle). The expression of JHDK in *L. decemlineata* was detected during all developmental stages, but JHDK was highly expressed during 1–3 instar larvae (Fu et al., 2015). This expression profile is similar to the profile observed in the present study, where JHDK expression was high during the larval stage. The knockdown of JHDK resulted in impaired adult emergence (Fu et al., 2015). Almost all of the identified genes associated with the JH pathway in the present study were expressed in the same manner; they were the most highly expressed during the larval stage, followed by the pupal stage, with the lowest expression during the adult stage, as demonstrated by real-time PCR results and FPKM values. Studies in many insects have reported that the genes involved in this pathway are also expressed in a coordinated manner (Kinjoh et al., 2007; Ueda, Shinoda & Hiruma, 2009; Nouzova et al., 2011; Huang et al., 2015; Verlinden et al., 2015).

During molting hormone biosynthesis, a steroid precursor is converted into an active ecdysteroid, 20-hydroxyecdysone (20E), a key hormone that controls physiological and behavioral changes related to molting, in a process that involves the Halloween gene family (*spo, spok, spot, sro, phm, dib, sad,* and *shd*) (Niwa et al., 2010; Song et al., 2017). During the specific rhythmic process of 20E biosynthesis, the initial conversion of cholesterol into 7-dehydrocholesterol (7dC) is achieved by the enzyme 7,8-dehydrogenase (Nvd). However, Nvd was not able to be identified in either the *S. aquatilis* transcriptome obtained in the present study or that for any of the other three firefly species that have been sequenced (Wang et al., 2017). Likewise, in the transcriptome study of the mealybug, *P. solenopsis*, no putative Nvd homolog was identified (Arya et al., 2018). The *nvd* gene was originally identify in *B. mori* (Niwa et al., 2004; Yoshiyama et al., 2006). Nvd functions as a cholesterol oxygenase and is conserved among insects and vertebrates. However, in *Drosophila pachea*, the Nvd ortholog has undergone several amino acid substitutions, abolishing the catalytic activity for cholesterol. Instead, lathosterol, a sterol from the host cactus plant, was used as a substrate for the Nvd enzyme in this species (Lang et al., 2012; Perry, 2018). Therefore, cholesterol may not act as an ecdysteroid precursor in the firefly; thus, a different enzyme may be required during this first step of ecdysteroid production. The next step in the ecdysteroid pathway is comprised of multiple conversion steps, described as “Black Box”, which turn 7dC into diketol. In the present study, three putative enzymes in the black box, Spo, Spot, and Sro were identified during the transcriptome analysis. Diketol is then converted into 3 β-5 β-ketodiol and into 3 β-5 β-ketotriol by Phm. 3 β-5 β-ketotriol is converted into ecdysone, and 20E by Dib, and Sad, respectively. The catabolism of the molting hormone depends on the ability of Cyp18a1 to convert 20E into an inactive form, 20,26-dihydroxyecdysone (Rewitz et al., 2006; Yoshiyama et al., 2006; Yoshiyama-Yanagawa et al., 2011; Song et al., 2017; Lin, Yu & Smagghe, 2018). The expression profiles for molting hormone-related genes identified in *S. aquatilis* demonstrated that most of the enzymes in this pathway (Dib, Spot, Sro, Shd, Phm, and Cyp18a1) were highly expressed during the larval stage (Figs. 6 and 7B). The final enzyme that degrades 20E, Cyp18a1, was identified in all stages, with a specific contig that was expressed highly in each individual stage (Fig. 6).

High-throughput sequencing technologies have facilitated the acquisition of the transcriptome data from different stages of firefly development. The present study revealed the gene candidates proposed to be involved in the insect hormone biosynthetic pathways; however, the functions of these candidate genes remain to be elucidated. These proposed pathways not only narrow down the number of target genes that must be characterized but can also be used to predict other related pathways associated with other developmental processes in the firefly. The present study is the first step toward understanding the molecular basis of firefly metamorphosis and will hopefully lead to an understanding of the regulation of the firefly life cycle, ultimately improving the firefly rearing techniques for both conservation and research purposes in the future.

## Conclusions

In the present study, candidate genes involved in the insect hormone biosynthetic and degradation pathways of an aquatic firefly, *S. aquatilis,* were identified through transcriptome analysis using an NGS technique. The expression profiles of these candidate genes were compared among the three stages of the firefly life cycle: larva, pupa, and adult. The expression profiles for most genes were consistent between transcriptome and quantitative real-time PCR analyses. RT-PCR and sequencing confirmed the expression of CDS expression of these putative genes. The present study is the first to analyzed the JH and ecdysone pathways in firefly, providing further insights into the molecular control of firefly metamorphosis. However, the functions of these gene candidates must be elucidated using other gene analysis techniques, such as gene knockout. Understanding the key genes involved in the firefly metamorphosis will allow us to alter the life cycles of these fireflies, an approach that could be beneficial for firefly conservation programs, the ecotourism industry, or biological pest control programs in the future.

##  Supplemental Information

10.7717/peerj.7428/supp-1 Supplemental Information 1 Quantitative real-time and RT-PCR primers

10.7717/peerj.7428/supp-2 Supplemental Information 2 Gene annotation

10.7717/peerj.7428/supp-3 Supplemental Information 3 Comparative analysis of unigenes among four firefly species

10.7717/peerj.7428/supp-4 Supplemental Information 4 Quantitative real-time PCR

10.7717/peerj.7428/supp-5 Supplemental Information 5 RT-PCR sequencing result

## Additional Information and Declarations

### Data Availability

The following information was supplied regarding data availability: The clean reads of nine specimens of *S. aquatilis* in this study are available at NCBI SRA: PRJNA525262.
