# Supplementary material for: Reconstruction of insect hormone pathways in an aquatic firefly, Sclerotia aquatilis (Coleoptera: Lampyridae), using RNA-seq
Source: PeerJ. 2019 Aug 2;7:e7428. doi: 10.7717/peerj.7428 (PMC6681800; doi:10.7717/peerj.7428)
Supplement: Supplemental Information 5 [file peerj-07-7428-s005.docx]

**RT-PCR Sanger sequencing**

>CYP15A1_CL9077_Contig2_RTSEQ_1521BP

ATGATAGCATTTATACTAGTTTTACTCATTGTGGTTTCGCTCAGTGTTATCTACTTTCATCAGCTTGCTAAAAAGAAACCAGAAAATTTTCCACCAGGGCCTCCGACGCTGCCTATATGGGGAGGATATTGGTTTTTGCTTAAGGCACATTATTATCTAACACACAAAGCAGCTGAGTACTTGAGAAAAAAGTACAACTCAGACGTAGTTGGTTTTTATGGAGGCGATTTTCCTATTGTTATTGTTACTAGTCATGCTTTGGTTAAGCAGGCTTTAAATAAAGATGAATTCATTGGCCGCCCCGATATATTTGCGACCAGAAACAGAACTCTAGGCGATCTCTTAGGGATTATATTCACTGATGGACCGCTGTGGAAAGAGCAGAGACGTTTTTCTTTGCGTCAACTGCGTGATTATGGTTTTGGTCGAAGATTTACCGCCACTGAAGATTTATTTGAAAGAGAAGTTAAATCATTATTGGAATTCCTTCACACAGACCCTTCTCCAAATGACTTGGATGTGTGCTCAAAAAAAGGCCGTGTATTAGTGCCAGACCTATTTTACGGGCTTCTCTCCAATGCCATTTTGCACATGCTAGTTGGATACAGGTTTGACGAAAAAGAACTAAGAGAATTAGGACGATCTTCAGTGAGATTTTTGCAAAACACTGATACAACAGGCCGCGCCCTCAGCATCACACCTTGGATTAGATACTTTGCTCCAAAGTTCTTTGGTTCAACTCAAATATTTGAAGAGAACAAAAAAATCAGAGACTTCTGTCAGAGAAGTATTGATCAACGGAAATTAACCTTTTCGAACGACCACAAGACGGACTTTTTAGATTCGTTTTTCAGTAAAATGCAAGAATTACAGAATGAGGGTTTAGATCTTGGTAGTTTTACAGAGAAGCAGTTGGTTTGGACTTTAGTAGACTACTTATTCCCGGCTCCTAATGTCATTGGGCCTTCTCTAAATATGTTATGGATGCATTTATGTATGTTTCCAGAAGTGCAAGTAAAAGTGCAAGAGGAAATTGATCGGGTTGTTGGTAGATCTCGCTTACCAACGCTGGATGATAGAAAAAACATGCCATATACAGAAGCTGTAATTCGTGAATCTTTACGATTTGATCCGGTAGTTCCTATAAACACACCGAGAAAATGCTTAGAGGACACCACATTTGGAGGATATTTTATACCAAAAGGCTCGTTATTGCTGATTTCGCTGTGGAACGCCAACTACGATTCAAAAGTGTGGGATGAACCTGAAGTATTCAGACCAGAACGATTTCTTGATCAAGACGGTCACTTGTTAAAAAAAGACAATATACTTTCTTTCGGTGCTGGAAAAAGATTATGCGCTGGCGAGACGTTCGCAAGAAATTCGATGTTTTTGTTGTTGTCTGGGTTGTTACAAAACTACACGTTTAAACCGGTACACGGAGTACCTGACAGAGAAAGTAAACATTGGGGTTTTATTGTTGATATTCCTCCTTTTTGGGTAGACGCTGTAAGCCGTTGA

>JHE_UNIGENE14277_RTSEQ_1245BP

ATGTTGTGCTTGTTGTGCCAAATTACAGACTGGGAGCGTTGGGTTTTTTTAAGTACCGGTGATGAAGTGTTACCTGGAAACAACGGCATGAAAGATCAGAGTTTGGCATTAAAATGGGTTGCAAATAACATATTGAAGTTTGGAGGGGATCCTAATAAGGTGACTATTTTTGGAGAATCTGCAGGCGGCGTTAGTGCTCACTTACACATGAAGTCGCCGTTAAGCAAAGGACTCTTTCATGCGGCAATTTCTCAGAGCGGCACGGTTCATGTACCTTGGGGATTAGCAAAACCAGGAGAGAATATAAAACAAGCTCAAAAGCTAGCAAAATCTTTTAACTGTCCAATTTCAAGTAGCCAGAGAATGCTTGATTGCTTGAAAAGTATTGATTCTTATGATTTAGTTGCCAAAGACAAAATATTTATGGAATGGGATACAGATCCAATGATTCCGTTTAGACCAGTTGTTGAGCCAGACTTACCTGGTGCATTTTTGTCAGAACATCCCAGCGAGATAATAAAGTCTGGAAAGTCATCACCAGTGCCGTGGATGACAGGAGTGACTACCGAAGATGGCGCATTGAGAGTTCCAGCTATATTTGGTAACCAACATTTGCTTCGGGAGTTGGATGAAGAGTTTAATCGATATGCTCCAATATCTTTGTTCTATAAAGATACCTGTACCAATCAAGACTATGTGTCTGACAAAATCAGAAAATTTTATTTGGGATCAAAAAAAATTGACAATTCTACACGGTATAATATTATAGATATGTATACTGATGGGTGGTTTATGCACGGTGCAGATGAATCTGTAAGACAACATTTGAAGTACACCTCCCAACCAATTTATTATTATTTGTTTGGACACCGCGGTGTTGCCAGTTTTAGCCAAATTTTTGGCGACCCACATCAACGTTATGGCGTATGTCATGCTGATGATTTACAGTATCTATTTCCTGTAGCAGATACCCTGTTTCCAGATCAAAAACAAGATGAGCTAGATAAGCGCGTAACTAACATAGTAACGACGCTTTGGACGAATTTTGCTAAATACCACGATCCAACACCTACAGTAGACTATATTTTACATGCAAAATGGGAACCAGTTAAATCAGAAAAGTTTGAATATTATTACATTGGTGGTGGTGTCCACTCAGGCAATGGTTTGTTGTGGGAAAGAGCACAGTTCTGGAGAAATTTGCCGATTAAATGGAACACTCAGTGCTTAAAAGATGAACTGTAG

>JHEH_UNIGENE8839_RTSEQ_1389BP

ATGTCGTTTCTTAGTAAATCTTTTTTGTTTATAACAACGGTGACGGTGGTTTATCTGGGTATAAAATTATACCCATTGTTCACAATTCCTCCATTACCCGATTTAGACGAAAATGCTTATTGGGGACCAGGAAGCCCACCTACCAAACAAGATACATCTATTCGTCCATTTAAAATTAACGTGACAGATGAGGTGTTGAAAGATTTGAAGTACCGTTTAGAAACAGATCGTCCTTACACTCCACCCTTAGAAGGCATCCAACAACAATATGGATTTAACACAAACCTACTCAAAGAAATTGTTGAATATTGGAAAACTAAATATAACTGGCGCGAACGAGAAAAATTCCTCAACCAATTTCCTCAATATAAAACAAAAATTCAAGGACTGGACATGCATTACATACACGTAAAACCTAAAGTATCACCACAAACTAAAGTACTTCCTATGCTGCTCCTACACGGGTGGCCGGGATCTGTTCGAGAATTTTACAAACTCATACCGCTTCTAACTAAAGAAAGCAAGGATCGCGATTTTGTATTTGAAATTGTCGCTCCTTCTTTACCGGGCTATGGATTTTCGGAAGGATCATCAAAAACAGGCCTAGGTACAAACGAGATTGCAGTGATTATGAAGAATTTAATGGCACGTCTGGGTTTTGATAAGTTTTACGTAGAAGGTGGCGACTGGGGTTCTGTTATTGTTACTGATATGGCAATATTGTTTCCAAAAAATATCTTGGGATTGCATTCAAATTTATGTAGAGCTTCAGGATTTAAATCTTTTTTAAAAGTGTTAGTAGGTAGTTTATATCCTCCTTGGTTTATTGAAAAAGAATACGAACACAAAATGTACCCTTTGACTTCACATTTCTACTTTTTAATAAGCGCATCGGGATATTTCCACTTACAAGCTACTAAACCTGACACTCTCGGTGTTGCAATGGGACAATCGCCTAGTGGTCTTGCCACTTACATCTTACAGATGTTTTCGCTTGGTACTAATACAACTTATATTGCCAGAGAAGATGGAGGTTTAAAAGAAAAATTTACCTACGACGAGCTTTTAGATAATGTCATGATTTATTGGATCACAAATTCATTTACAACAGCTATAAGACTGTACGCAGAATGTGGTCCAAAGGCTTTTACTCTCGCGACAACAAAAATACCAATAAGCGAAGAAGTTCCGTGCGCTTGTGCAAGAATGGGATTTGAAATTAGTTATTTTACCGATTGGATTCTAAAAGATAAATATCCAAACTTGATCCAATCTACGGATTACAAAGTAGGTGGACATTTTGCTGCAATGGAAGAACCTAAACTTTTTGCAAACGATATATTGATGGCTGTTGAAAAAATGGAAAAAATAAGAAACATCAGAAAATAA

>JHEH_UNIGENE9157_RTSEQ_1383BP

ATGGCGTTCCTCCGGAAAGTGTTTTATCTTATTCTTACGTCTTTTATCGTTTATGTAGCGTTTCAAATCAATGCATCATTAGAAATACCTCCAATACCCAATTTAGACAACGAGTATTGGGGACCCGGTTCTCCTACTAAAGATGACATCGCAATAAGACCGTTTAAAATTAACGTTCCCGAACAGGTTCTAGAAATTTTTAAATCTAAGTTAAATCAACCGCTTGCATTAACACCACCGCTAGAAGGAATCCAACAGCAATATGGAATGAATACAGAGTTATTAAAATCAATAGTAGAATATTGGAAAACTCAATACAATTGGCGCGAACGAGAAAAATATTTAAACACGTTTTCTCAATTTAAAACCCAGATCCAAGGCCTAAGCATTCACTTTCTTCACGTGAAGCCCACCGCGACAGATGGGAAAGAAGTCATACCTTTACTTTTAATTCACGGCTGGCCTGGATCTGTCCGCGAATTTTACGATCTAATTCCACTTTTAACCACCCCTCAGAAGAATCATAATTTTGTTTTTGAAGTCATAGTACCTTCGCTGCCTGGATATGGATTTTCGGAAGGATCGTCAAAACCCGGTCTTGGATCAGCACATATAGGTGCTATATTTAAAAAACTAATGCTTCGATTAAGTTTTAATAAATTTTACGTTCATGGCGGTGACTGGGGAGCTATGATTGCACAGTTCATGTCTGGTTTTTATCCAGAACATGTATTAGGAATGCATTCTACTATGTGCTCATCAAACACATTATTATCACAATTGAAAATGTTTATTGGTAGTGTTTATCCTCCTTTAGTAGTAGACGAAAAACATCAGCACCTAGTATATCCACTGAGCAAATTGTTTTCAAACATAGTTTTGGAATCTGGTTACATGCATATACAAGCCACAAAACCTGACACTATTGGTGTTGCTTTGAGCGACTCGCCTATAGGGTTAGCTGCGTATATACTAGAAAAGTTCACAACATGGACCAATCCCGAATGGAAAAATAGAGCTGACGGTGGTCTATTAGTGAAATATAATTATACAGATCTCTTAGACAACGTTATGATTTATTGGGTAACTGGAAGTATTACAACTTCCATTAGATTATATTCAGAAAGCTTAAGCAATAAGTACCTTAATAATCATTGGGAAAGAATACCAGTTCAGGTTCCTACAGCTTGTGCTAGATCCGATAAGGATTTATTCTATACACCAGACGCATTATTGAAGGATACATTTAAAAAACTAGTTCAAGTTAAGGATTTATCAGAATGTGGCCATTTTTGTTCTTTTGAAGAACCGCAGGTAATAGCAGGTGACATTTGGCCTGCTGTAGAAAAATTTCGAGAAGTTCATAAGAAAATTAAGTCTTAA

>JHDK_CL987_RTSEQ_549BP

ATGGCAGATTTCCGCAAGAAAAAACTACTTTACGTTTTTAACGTCTTTTTTGATGTAAACCAAAGTGGGACTATTGATAGAAAAGATTTTGAGTTAGCTATTGAGAAAATTTGTAAGTTGAGAGGATGGGCTCCTGGAACTGAACAATACAAAAAAACGTTTGATAGCCTTATTCAAATTTGGGATGGACTTAGAAACCGAGCTGATGCTAACAAGGACGGACAAGTTAGTGTGGACGAATGGTGTTTAATGTGGGATGACTTTGCTAAAGCACCTAACAATCCTTTGGAATGGCAAACTCAATATCAAAAATTTATGTTTGATTTAGAAGATGCTAGTGGAGATGGTGCTATTGATGCTGAAGAATTCACCAGCGTTTGTTCTTGCTACGGATTAGAAGCTTCTGAATGCAGAAATGCTTTCCAGAAGATGTCTTGTGGAAAAGCCAGTGTTTCTTACGAGCAGTTCCAATCTTTGTGGAAACAATATTTTGTTTCTGAAAATCCATCTGATCCTGGCAACTATATTTTCGGCAAAACCAATTTTTAA

>PHM_UNIGENE12481_RTSEQ_1455BP

ATGATGATCTACATTTTGATCGCGTTGTTGGTTTTGACTTTGACCTTTTGGTGTCTGCAAAGAAAGCTACCGCCTGGTCCGTGGGGTCTACCAATTCTTGGTTATCTACCGTTTTTAAATCCGAATGCTCCACACGAGACGTTAACCGAACTAGCGAGAAAATATGGAAAAATTTATGGGCTACGACTGGGAAGCATTTACACTGTTGTTTTATCCGATCCTGCAATGATTCTTAGTGCTTTCTCCAAGGATAAGCTTACAGGACGAGCTCCTCTTTATGTTACTCATGGTATTATGGGTGGATATGGTATTATTTGCGCTGAAGGTAATATGTGGAGAGTACACAGGAAATTTACCGCAAAGTGTTTGAGGTTATTTGGGGCAACTAAATCTTCTGGTTTGAAAATGCAAAATTTGGAGGAATTAATTATGAAAGAGGTTCAAGACTGTCTAAGCAATCTTAGGGAAGATTTAACGGTGGATCCTGCTAAATATCTTCAACATAGCGTAGGTTCAATTATGTGTCGTTTGGTTTTTGGCAAGGCTTGGAGTAAAGATGATCCCACGTGGCTTTGGCTGCAACACTTGCAAGAAGAAGGAACTAAGTTGATAGGCGTAGCTGGTCCGCTTAATTTTTTGCCGTTTCTGAGATTTGTTCCCGCCTACAAGAAGGCAATGCACTTTTTACTCGACGGCAAACATAAGACTCATAAGATTTACGAGGAACTGATACAACACCAAAAACAAACGTTTGAAGTAAAAGGCGATGCTAATCCCGAGAACGTAATACAAGCGTTCTTACGTGAGCTGCAAACAACAAACGATGCAGAATATTTCACCAATGAGCAATTCTACCATCTACTTGCAGACATTTTCGGAGCAGGGTTGGACACAACGTTAACTACTTTACGTTGGTTCTTTCTCTATATGGCGGCTTACCCAAACGTCCAAAAAGAAATCCAAAAGGAATTAGACACTGTTCTTATTAACCGTCTACCAACGCTTAAAGATGCCCAAGTACTACGTTTGACGCAAGCAGCCATCGCCGAAACTCAACGTATTCGATCAGTTGTTCCACTTGGTATCCCACACGCAACCACTGATAGCATTGAATTATTTGGTTATTGCATCCCCAAAGGCACCATGGTCGTACCCTTACAATGGGCTATTCACATGGACCCAAACACGTGGAAAAATCCCCAAGATTTTAACCCCAATCGGTTTTTAAACGAATTCATGCACTTCTCCAAACCAAAGGAATTCATTCCTTTCCAAATTGGTAAACGCGTGTGCTTGGGAGAAGATTTGGCCAACATGATCATGTTTCTGTTCACCTCGAGCATCCTCCAACAATTCGAAGTGTCAAAACAAGAAGATCAAGTGGATTTGGATGGTGAAATTGGTATTAGCCTCACGCCCAAGTACCAAATTCTTTCTTTTAAATTAAGATCCTGA

>SAD_CYP315A1_UNIGENE15996_RTSEQ_1422BP

ATGATTTGCCGGTTCAATAAAATCACGCACGCACACGTATTTAAAATATCACCAATTAGATGTGAAAGTTCGGCCACGGTGCTGTTAGATTATCAAAATATACCGACGGCGAAGGGGCTACCTCTGATAGGAACGACGCTAGCCCTAATAAGTGCGGGAGGTGCGCCTCGTCTACACATCTACGCAAACAGGCGACACCAGCAACTCGGACCAATTTTTAAAGACAAAATCGGACCCGTAAGTGCAATTTTTTTATCCGATCCCGAATTAATGCGCTCGGTTTTCGCGCAAGAAGGAAAATGCCCGATACATTTGCTACCCGAAGCTTGGAGTTTGTACAACGAAAAAAACGGCTGCTCCCGCGGGCTGTTTTTCATGAACGGGGAGGAATGGCTCAACAATCGCAGGATTATGAATAAGTTGTTGTTAAAAGGCGACACACGATGGATCGAACGTGCCTGTGAAATCGTAAGCGAGGATTTAGTGCAACGCTGGAAAAAATTAACTTCTCACGAAGAAGTACTTCCACAACTGGAAGCGGAACTGTACCGATGGTCCCTAAACACAATCGTCGCCGTACTATTGGGAGAAAACGACTACGAAAAGCAAAAGACTGAACTCGATACCCTCTTAGAGGACCTGTCAAGAACCGTCCATTTAATCTTCGAAACCAGCGTTAAATTCCAGGTGTTCCCTGCTACAATTGCACAAAAACTGGCGCTACCCTCTTGGAAAAGGTTTTCGAAAACCGTCGACAATGCTTTGCGTCTAGCCAACGAATTACTCGCCGTTTTAAGAAAATCGAGTTTGGACGACCAAGGGTTGTTATCGCGAATGCACCGGGAGGCCATCGACGAAAAAGACATCACGCGCATTATAATTGATCTGGTTCTGGCCGCCGGTGACACAACCGCTTACACCATGTCCTGGATTTTATACTGCTTAGCAAAAGATCCCCAAGTGCAACAAAGCGTGCTCGAGCGTAGCAACCAAACGACGCACTTAAAGAACGTCATCAGAGAGACGCTGCGTTTGTACCCGGTCGCGCCGTTTCTAACCCGCGTGGTTCCACAAAACACTTTAGTAGCTGGTTACGAAATACCGGCAGGTACCTTAATGATTTTCTCGATTTACACCAGCGGACGCGATCCCAGATACTTCCAAAATCCGAACGTCTTCCATCCTGATCGTTGGTTACGCGAAAACAGCACGGCGAACATGCAACAAGTCTGCATACCCTTCGGATTTGGTGCGCGCTCGTGCATCGGAAAAAAAATCGCCGATTACCAGCTCCAAACCTCCATAGCAAGAATTCTCCAAAATTTCACTCTGGGTTTGGCCAACAAGGAGGAAGTTGACATGGTCTTAAAAATGGTGGCCGTGCCTTCGAAACCTATAAGACTGAAGCTCGATCGTATCTAG

>SHD_CYP314A1_CL7713_CONTIG2_RTSEQ_1479BP

ATGCTGTTTGACATTTCACGAATTCAAGATGTTTTTGCGATATTCGCTTTAATAATTTTATATCTGCTTGTTGGATATCGTCCACCTTGGTTATGGAAACACTATGAGAAAGATCGAAAAACCATAAATACTGTTCCAGGACCTTTTTCTTTGCCTTTTATAGGCACTCGTTGGATTTATTGGTTTGGCAATTACTCTTTTACAAAAGTACATGAAGTATATGCAGATTTATTTAAACGATATGGTTCAATTATTAAGGAGGAAACTTTATTTAACATTCCTGTTATTAGTATTTTAGACCGTAACGATATTGAAAAAGTTTTAAAATCTAGCGGAAAGTTTCCAGTAAGGCCTCCTACAGAAGTAATAGCTTATTATAGAAAAACTCGTCCTGATAGATATGCTAGTGGTGGACTTGTTAATGAACAGGGTATGGTATGGCATCATTTACGCACAAATTTAACTTCAGAACTTACTAGTCCACGAACCATAGCAAGCTTTATACCTCAAATTGATGAAATAGTTGAAGAATGGTGCTATTTAATCAAACAAATGAGAATTGAAGGAAAACAAATTGATGACCTAAAACCTTTAGCTGAAAGGTTGGGGCTTGAAGTTACTTGCGCTTTGGTATTAGGACGTCGAATGGGATTTCTGTTGCCTGATGGTATATCACCAAATGCTCAAGCATTGGCAGATGCTGTTCATCAACACTTTTTAGCATGTCGTGACACCTTTTATGGACTGCCATTTTGGAAAGTATGGACAACTCCTGCATATATGCATTTGGTGGAAGGTGAGGAAGCAATTTACACTTTTGCATTGGAATTAATTGCTAGTGCTAATGAGGAAACCAAGGAAAGTGTTGTTTTTCAGTCTGTTTTGAAAGCTTCTGTGGACGATCGAGAAAAAACTGCAGCAATAGTTGATTTTATAGCTGCAGGCATTTACACTTTAGGCAACTCAATTGTATTTTTGTTACATTTGATGGGCAGTAATCCAGAATGTCAGAAAAAATTGATTGAGGATCTCAACAAAGGTTCTACTACTTATCTTAAGGCTTGCATTAATGAAGCTTTTCGATTGATTCCAACAGCATACTGTTTGGCTAGAGTCTCTGAACAAGATCTGGAATTGTCAGGTTTTCACATTAAAGCAGGAACTGTTTTGTTATGTCATACGGGTTTAGCATGTAAGAATGAAAGTAATTTTAAAGATGCTTGTAGTTTTAAACCTGAACGTTGGATAGAAGAGTCGAAAGCAAGTACTATATCAACCGCAACCTTTTTAGTAACACCTTTTGGTGTTGGAAAACGTATATGTCCAGGTAGAAGATTTGTAGAACAAGTTTTAACCTCTTTGCTAATCAATGCAGTGGAACAATTTATTATCTCGCGTGACAATGATCTAGAGTTACAATTTGAATTTATATTAGCTCCAAAGGGACCAGTAAAAGTCTTTTTTGAAGATCGCCACTAA

>DIB_CL5335_CONTIG10_RTSEQ_1506BP

ATGTTGTATTTTAACACACACAAAAAAAATATTTTTATACGTATTTTAAGCACTCGTTATGGGTCCACAAAGAGCTTCCTTCACATTCCAGGACCTTTATCACTGCCAGGTATTGGTACATTATATCAGTATTTACCTCTTATGGGTCCTTATAAATTTGACCGATTACACTGGAACGGTTTCAAAAAGTTGAAGAAATATGGTCCAGTTATTCGCGAACAAATGGTACCTGGTGTTAACGTTGTTTGGCTGTTTAAGCCTGAGGATATTGAGATAATGTTTCGTTCTGAAGGGAAATATCCTCAAAGGAGGAGCCATCTTGCTTTAGAAAAGTATCGTTTGGATAGACCCAATGTGTATAACACTGGGGGTCTTTTACCAACTAACGGTCCTGATTGGTTAAGGTTAAGAAGTATTTTTCAAAAAGGTTTAAGCAGCCCAAGTGCTGTACATAACTTTTTACCTGAATCAAATGAAATTATTCAAGAATGGTTGTACAGATTGGAGGAAATATGTAAAACACCAGATTTAATTTATGCTAATGAACTGTCTCGATTGTTTCTAGAATTAATTTGTCTCGTTGCTTTGGATTTGCGAATGAACAGCTTTACCAAAAGGGAATTACGACCCAATTCTCGATCGTCTAAATTGATGAAAGCTGCTTTAACCACAAATAGTTGCATTTTAAAGACTGATAATGGACCACAACTTTGGCGTAAGTTTGAAACTCCGTTATACAAGAAATTGCGAAAATCTCAGGAATTTATGGAAGAGGTAGCTATAGATTTGCTCTCATTAAAAATGTCTTTCTTTAAGGAAGACAATAAAAAATCAGGATCATTGCTGGAGAGTTATTTGTCTTGCCCTGATTTAGATTTTAAGGATATTATAGGTGTTGTCTGTGATTTTCTACTGGCAGGTGTTGACACAACTTCTTATACAACTAGCTTTATTCTGTATCATCTGGCAAAGAATAGGCTTAGCCAAAGCACGTTATTTCAAGAATGTAGAAGACTTTTACCTAAGCCAGATAGCCCTGTGACTAAGGAGGTTTTAAGTCAAGCTCAATATGCAAAGGCTGTTATAAAAGAATCATTACGTTTAAGGCCGATATCAATAGGGATAGGACGTGTTCTTGACAAAGAAGCACAGTTCTCTAACTTTACCGTTCCACGTGGGACTGTTGTAGTTAGCCAAAACCAAGTATCTTGTCGTTTGAAAGAATATTTTACAGATCCAAATGAATTTAGACCTGAAAGGTGGTTGAAAAATCACAAGTACTATCAACAGCCACATCCATTTCTGGTAATACCATTTGGACACGGTGCTCGTTCTTGTATTGCCAGAAGGTTGGCTGAACAAAACATGCTTGTATTTATTTTAAAGTTAACCAGAAACTTTAAATACCGGTGGGATGGATCTGATATCGATACAAAATCTTTCTTGATAAATCAACCAGATGGACCAATATCATTAATAATGGAAAAACGTTCCGAAGATTCATGA

>SRO_UNIGENE10748_RTSEQ_1110BP

ATGGGTTCGAACTTTTCTTTATCTTCAATTACACTGACTGCTTTTGAAATCATAAATGAAATTTTTATTGCCGTTGGTACTGGATTGACCGGTATTGTAATTTTGCTAAATCACGGTGTGAAAAGCAACGAAGTTTTAAAAACGGTTCTTATTTCAACTGTGACGATAAGTTGTTTGTTTTTTTACTTTCAATCAAACGAAAGTACAGAACCATCAGCTGATAAAGTTGTTTTTATTACTGGATGTGATTCTGGCTTGGGTTATTCGTTTGCGCAACATCTGTGTGAATTGGGTTTTACTGTTTTGGCGGGTTGTTTAAGTTTAGATTCTAAAGGGGCACGAAAATTAAGGTCTTTATTTGGAGATAAAATTAAACATATTGAATTGGATGTTACAAGATCCACCAGTGTTGAAGTGGCCGTTGACGTTGTAAACGAAATTCTTAAAACTCACCCGAATTATGAACTATGGGCAATAATCAACAACGCAGGTGTTATGGTTTTTGGCGAATTTGAATGGTTAACTGAGAAACTTGTTCAAAAACAACTTGATGTTAATCTTTCGGGTACGTTTAGGTTCACAAAAGCATTTTGTCCTTTACTACGCCAACACAAAGCTAGGCTTATCAACATTTCAAGTCACTGTGCTTTAGCGTCTCTTCCAGGCTTATCAGTGTACGGAGCTACCAAAGCAGCTCTAAAAGGTTGGAATGACGCTCTACGAGTGGAGTTGAACAAATATGGAGTGGATGTTATTCTTTTTGTGCCGGGTTCGTTTATACAACAAAGTAATATAATGGCATCACAAGTGGAAAGTTGTTTTGAAATGTATAATGCTTTTACGAAAGAGCAATTACAATTTTATGGAGATTATTTTAATCGGTATAGCAACTACTTAAATGTGTTGGCTGGTCCCAAAATTGTTGAAAAGATTGACGACGCTTATCTATATTTTAAATTAGAAAAAGCTTTATTGGACATGCCACCATCGCCTGTATACATTCACGAAACATTTTACTATAGCGTGTATCACATGCTTTTTAAATACAGCCCTATTAGAGTTAGAGATTATTTAATCAAGCGGTTTATGCAAATGCCTTCCTATATTTAA

>SRO_UNIGENE21598_RTSEQ_1182BP

ATGAGTGGACCGTTTAAAAGACGAGCTTCTCTTAAGAAGCCGGTTTCTTTGGGTTTAGTGAAATCTGCAGCTACTCGTAGGAGGTCGTCCGTTACAGCTTTAGCACCCATTCCTCAAGTACAACAAGATGTTCCGTGGGATCTTCTAGAAAGATGTTTTTTACCTGTTCTTTTTTGTCACGCTGCCGCTGTAGTTGTTAGTTATACCCTTAGTGTACTTCGAATATGGCAAGTAACAGCGTTTTCGTTATTTATATGGTTTACAATAGTTATGCTGGGGGTGGTGCTGTTTTATCACAATCTAAAGGTTGCTACAGTCGGGAAAGCAGTATTAATAACTGGTTGTGACTCACGAATAGGAAGTGCTCTTGCCAGAGTTTTAGACGATCTTGGATTTACTGTCTTTGCAGGATTTCAAAATGCGATGGATAATCCGGTTGCAAACGAATTAAAAGAAGAATCTTCAGGCCGTCTACATATTTTACAATTAGACGTATCATCAGAAACACAAATTTTAGCAGCATCTCTATACGCAACTGCACATTTACCAGACGGCGCCGATGGTTTATGGTCTGTAATACATGCGGCTTCCTGGATTGCTTTAGGAGAAATTGAATGGATTCCTGTTGACATCATACGAAAAGCTACTGATATCAATTTTATTGGCGCTACCCGTTTAATGCAAGTCATGCTACCTTTAGTAAGAAGAGCAAAGGGTCGTATTGTTCTTGTAACCTCAGGACTTTCAAAAGTTGTGTCACCTGTACGTGGAGTTCATTGCGCACTTCAAGCAGCGTTAGAAGCAGAAGCTGTTTGTCTCAGACAAGAATTAAAACCCAGAGGAGTTGATGTTATTGTTGTTGCACCAGGAGAATACTCATCGGGAAGCTCTTGGGTAACTGAAGAAGGAATTCGCGAACAAGCTCGAGAAATGTGGGAACAGCTAATGGAAGAACAAAGACTCGAGTATGGAGAAGAATATTTTGAAACCGCTGTAAGAAGTTTAGAAAAATACACAAAATCTAAGGATGCTGATTTATCACCAGCACTTCGTGCTTTATCCGATTCGGTAATAAGGACTTTCCCGTTACAAAGATACACACCGATCAACAGACAAGAAAAGGTTCAAGCAACAATCGCCACTTATCTACCAAGATCTATTTACGACATTATTTATTCATAA

>FPPP_UNIGENE19505_RTSEQ_915BP

ATGTCAATGAAAAATTTATTTGATTTAAATTCAGAAGATTTATCATCGTTCTTTCAATCTTTTGACATCGTTCTATGTGATTGTGATGGTGTTTTGTGGTTGGGGCACATACCAATACCTGGAGTAGAAGAAACGCTTTCTACTCTAAAAGAGTGTGGCAAAAAAGTTTATTTTGTCTCTAATAACAACACTGGAGGAGTAAAACATGTTTTAAATGGTTTGCACAAAATTGACGAAAATATCGAAGAAAACGATGTGGTCATTCCAGTACAAGCAATTATATCGTTTTTGAAAGAGCTCAACTTTAATAAGAAACTCTTTGTATTAGGCACCAAAGCTATGAAACAGAGTATCGTGGATGCTGGTTTTTCTTTGGCTGACTCTGAGAGTACAGACTTCATGGAGATGATACACACGTATAGGGACGATAAAAAACTAAATAAAGATATAGGTGCATGTATACTTGACATAGATTATGAGTTATCATATAAGAAGTTGACAGAGTTTATGGTTTATCTCAATGATAAGGATGTTCTTTTTATAGTTGGTGCTACTGATAGATACCTGCCACTTTCAAGTAACAAGACTCTAATAGGACCTTGGTATTTTGAAAATATGCTACAAGAAAACACTAATCGTACTTCGATGCAATTTGGAAAACCATCGTTGAACCTTAATAAATTCATATTGAAGAAGTATAATATTAAAGATCCTTCTAGAGTTTTGGTTATTGGAGATTCGTTGGATCAGGATATTCAATTTGGTATAAACTCAGGTTACCAAACGCTGTTGGTGTTAACGGGCGTTACTAATTTAAAACACTTGGAAAACTGTGCTACTGATAAAACTCCTGACTATTACATAAATAGCGTGGTTGATCTTAAATCCTTGATTAAAGATAAATTAGAAATGTAA

>FPPP_CL2037.CONTIG1_4_5_RTSEQ_918BP

ATGTCCTCAAGAGACTTAACCAGTTTGTCTGCCTCGGAGTTAAGAAATTTCTTGAATTCGTTTGACACAGTTTTATGCGATTGTGATGGTGTACTATGGAATGTTGGAAATTCCATACCAGGAGTGACAGCAGCTCTCCAAAAGTTTAAACAATGCGGAAAAACGGTCGGTTTTGTTTCCAACAACAACCTATACGGCACTAGAGGTCTTTACAAAACCATCAAAAAGTATGATAATGAAGTTGAAATGAAGGATGTTGTTGTACCTGCTCAAGCAATTGTTGCTTATTTCAAAAGTATTCACTTTGAGAAAGAGATATTTATATTCGGAAGTGCAGCTATGAAAGAGGAATTTGAAGAAGCAGAGCTAAAAGTCGCAAACAAAAAGATAAGTACAAGCAGAAACATAAATGACCTAGTCAAAGAATTAAAAGCAAATAATGAGAGTATTGGTGCAATCGTGTTTGATTACGACATCTTTATAAATTATTTAAATCTGGTACAAGCCGTCATAATTCTTAAAGAAAGTAACGTTATTTTCGTCACTGGTGCAACAGATGATAAAGTGTATATTGATGATAATTTAGTAGTACCAGGACCCCAATCGTTTTTAAACGCTTTATTAAGCGTCTGTGACAGAAATCCGATCCAATTTGGCAAACCATCAGAAAATCTAGCTAAATTCATCAAATTGAAGTACAACATTCAAAACACATCACGAGTTTTGTTTGTGGGAGATTCACTTGCACAAGACATTGAGTTTGGTGAAGCTTGTGGCTTTCAAACTTTATTGGTATTAACAGGAGTATCGACATTAGCTGATGTAGAAAACAGCAAAAATATAAAAGAAGTGCCAAATTATTATATCGATAGTTTATCAAGCGTTAAAGAAGTCATAGAGAATAAGTTAAGCATATAG

>FOHSDR_CL8545_CONTIG2_RTSEQ_765BP

ATGGTCGCGTCTATGTCAAGGTGGGTCGGAAAAGTCGCTGTTGTTACAGGGGCTAGTTCCGGAAGCGGTGCCGCAATCGTAAAACGATTGCTGGAAGAAAATGTCATAGTTGTAGGTATTGCAAGACGCAAAGAAAAAATTGAAGCTTTAAGTGACTCAAAAAACTTGCATGCTATAAAGGCCGATGTAACAAAAGAAGAAGAAATTTTGCAAGCGTTTAAATGGATTAAAGACAATTTGGGACCTATTCATATTTTGGTTAATTGTGCTGGTATAATCAGAATGACAAATTATCTTGACGGCGACACAAAACATTGGAAAGAAATGATGGATACTAATTATCTTGGTTTGTGCATATGTACAAGGGAAGCTGTTAAAGATATGCGAGCTAATAACGTTGATGGTCATATTATTCATATTAACGATATTGCCGGATACAAGTTAATAGGTGTAGAGCATATGAACGTCTATTGTGCGAGTAAATACCCAGTAACTGCTGCTTTAGAAGCATTGAGAGTTGAATTAAATGCAATCAAAAGTAAAATTAAGATTTCCGGAATCAGTCCAGGGTATGTAACCACAGAACTGCTCGATGCTTGTACTAAAGTCCAACCAGAAATGAAAAAGTTTTTTGAAGAGTTTGACGATGCAACTGTGTCTTTGTCAGCTGAAGACGTAGCTGATACTGCAATTTATCTGTTATCTACTCCTCCTCACATTCAGATCCATGATATAATCATACGACCTGTTGGGCAAGACTTTTAG

>FOHSDR_UNIGENE12692_CONTIG2_RTSEQ_750BP

ATGGTTTTGTCTATGGAAAGATGGGTTGGAAAAGTTGCTTTAGTAACAGGTGCGAGTGCTGGATGTGGCGCAGCTATTGTAACTCAACTGGTAGAAAATGGGCTTAAGGTTATTGGATTTGCTAGACGCAAAGAAAAAGTTGAGGAGATTGCTAAGTCCTTAGAAGGAAAACCGGGTAAGCTATACGCTTACAAGGGTGATATTACAAAAGAAGACGACATACTGAACTGCTTTAAATGGACTAAAGAGAATGTAGGTCCAATCAGTGTACTAGTAAACAACGCTGGAATTAAAAGAAACACCAATTTAACCAGCGGAGATACAGAGCTCTGGAGAGAAACTTTTGAAACAAACGTTATCGGTCTTTGCATTGCCACAAGAGAAGCGGTGAAAGAAATGCGGACTAATAACATTGATGGTCATATCATTCATATTAACAGCTTAGCTGGTCATAGAATCTCTACTGTTGTTGTTACAAACGTATATCCTGCCAGTAAGTTTGCAGTTACAGCCTTAACTGAAACTTTAAGGCAAGAACTGAACACCATTGGAAGCAAAATTAAAATAACTAGTATTAGTCCAGGAGCTGTTAAAACTGAGTTCAGACAAGCATCCAATTTAAAAGAGGACGAAGAAAAGTTAGCTAAGAAGCCTATTTTGGAAGCAAATGATGTGGCGGATGCGGTTACATATGTTTTGTCGACGCCTCCGCATGTACAAGTGCACGATATTTTAATTCATCCGTTGTAA

>ALDH_UNIGENE21948_RTSEQ_1494BP

ATGGAAAATCCGAAAGTTTTAGTAGAAGAGGTAAGAACGGCGTTCAAAAGTGGACGCACAAAATCTTTAGCTTTTCGAGAACAACAGCTAAAAAACTTAATTCGTTTCTTCAAAGAAAATAACGATGAGATTATAACTGCGCTACACGTCGACATGAAAAAGAATGAACATGAAGTCATATTGTTTGAAACTGAAGGTAGCTTGAAGAAAATAATGTACACTCTTGAAAATTTTCGAAACTGGGCAGAGTTTGAAAGACCACCCAAATTTCTTTTAAATTTGATGGATGAAGTTGTAATTTATAAAGAACCTTATGGTGTTGTTCTTATTATTGTACCTTGGAATTATCCTGTATTTCTCACTATGGCACCATTAGTTTCTGCTATAGCAGCTGGTAATTGTGTTGTTATTAAACTATCTGAAATGTCACTTGCAATTAGTAATTTAATGGTAAAACTGCTTCCAAAGTATTTAGACCAAAATTGTTACAAAGTAGTTATTGGGGAGCCTGAAGAATCCAGCCGCCTATTAGAACAAAGATTTGACTACATATTTTTTACTGGTTCTTCAAGAGTCGGAAAAATTGTACATGCAGCTGCTAGTAAACATTTAACACCAACAACTTTAGAAATGGGCGGAAAATGTCCTGTTTTTATTGATGATAACGCTGATGTCAGCATAGCAGCTCATCGTATCCTATCGGGAAAATTTGTTAACGCTGGACAAACCTGTGTAGCACCAGATTATGTATTGTGTAACAGAAGCGTTGGGCAAAAATTTATAAGTGAAGCAAAAAAGGTGATTAAAGAATTTTATGGAGAAGATGAATCTAAATCTTCTTTTTATCCGCGTATTATTAACAATATTCATTTTGATCGTCTGTCAAAATATTTAAAAATAGACAAAATTGCGTATGGTGGAAAATCTGATAAAAACGATCTTTTTATCCAACCAACAATCTTGGATGACGTTAAGCCTACTGACGATGTCATGCAGGAGGAAATATTTGGTCCTATTTTACCTGTTGTTTATGTAGAAAATGCCCAAAAAGCTATTGATTTTATTAATTCGAGAGAAAAACCCTTAGCTTTATACGTTTTTACAAAAAATAGAAGTATTTACAAATCGTTTTTGGAGAACACTTCTAGTGGAGGTGTGACAATTAATGACGTTTGTATGCACGCGCTAATTGATAACGTACCATTTGGAGGAGTAGGATTTAGTGGAATGGGTTGCTGCAACGGACAATACGGTTTTGATATATTTGTCCACAAGAAAAGTGTTCTTGTTAAGAAATTGAATTCTTTTCAAGAAAAATTATGTAGTTTACGTTACCCTCCTTATTCGGATTCTAAGACAAACAAAATAATCCAAGTGTTAAACTTTCGTACGCCTTTTTCAACCAAATACTTTTCGTATCCTTTAATTTTTTCGTTTGTTGTATTGTCCGCTATATCTTTTAAGTATATGATAAATTTTTACAATAAGAAATAG

>JHAMT_UNIGENE9200_RTSEQ_810BP

ATGAATTACCCAGAATTATACCTCAGTTTTGAAAGTTTTTCGAACCAAGGTTCAGAAGCACTTATTAAAAAGTACTTTAAGTTAGGTGGTTCAAAAAAAGATGGTGAAACTAACGTATTGGACATTGGGTGTGGACCCGGAAACATCACCCAAAACGTTGTTGTCCCGATCTTCAAAAATTGCGCTCGCAAAATGGTAGGCATCGACGTATCGCAAAAAATGGTCGATTTCGGAAACGCAACGTACGGCAACGACTGGTTTTCGTTTAAAGTGTGTGATCTCGAAAAAGAAACTCCGCAAGAATTCATTTCTTATTTCGATTACGTGTTTTCGTTTTGGGCCTTGCAATGGATTCAAGATCAAAGGCAACTGTATAAAAACATTTACAAAATAATGAAACCCGGGGGTCACATCTTCTTAACTTACATAGCGCAAAGCAAACTGTACGATATCTACCAGTCGGTTTGGCACAAACCGGAGTACGCCCCCTACATCACCAATTTTCAACAAGCTCAATCGTGCTTCCAAAAATCGAAGGATCCCAAAAAGGAGCTGGAGGTGTTCGTAAAAGAAGCCGGTTTCGACGTCAACATGATAAAAATAGAAACAGTTCAACTAAACAGCTCCTTCACTGACTTCAAAAACTTTTTAGCTTCTATCAGTCCGGTTTCACATCAAATTCCGGAAAATCTTAGAGAGAAGTTTTTGATGGATCATGTGCAAGAAGTTGATAGGAATCCAGGAGCTGAGCCTCCCAACGATTACTGCATGGTTTTTGATCTATTTGTTGTTTATGCTAAGAAAAATTGA

>CYP18A1_CL2776_CONTIG3_RTSEQ_1587BP

ATGTTTGTGTACGGTTTTTGGTTATTGTGGGGTGTTGTGTCGTCGCAAATATCGGCCAGTGTTTTAAGCGTATTTTTTGTTGTGTTATTAATTGTTCGAATTATACAAATGATCCAACAAACGCAGTCGTTACCACCGGGTCCCTGGGGTCTACCTATTGTCGGATCGCTGCCGTTCCTGAAAGGCGATCTTCATCTACACTTTCGTGACTTAACGCAAAAATACGGCTCTCTACTGTCTACCCGTTTGGGATCACAACTAATAGTAGTGTTAAGTGACTACAAAATGATAAGGGACGCGTTTCGCAAAGAAGAATTCACTGGCAGGCCGTCCACTGAATTTACTAGCATACTAGAAGGCTACGGTATAATTAACACGTCAGGAAAACTGTGGAAAGACCAACGAAGGTTTCTGCACGACCGTCTAAGACGTTTCGGCATGACTTACATCGGCGCCAGAAAAACGCAAATGGAAAATCGCATCATGACCGAAGTCGAAGAATTCCTGTGCATTTTGCGCGCCAAAAAGAACAGCCCGATCGACTTCAATCCGATTCTTGCCGTATCAATTTCGAACGTCATCTGCGACATCATAATGTCTGTGCGATTCTCGCACAACGACGCTCGTTTCCGCAGATTCATGGATCTCATCGACGAAGGCTTCAGGCTATTCGGTTCCCTAGAAGCGGCCGTCTTTATACCAATCTTACGTTACCTACCCGGGTTGCAAAAAACTCGCCAGCAAATCAGTAAGAACCGACAAGAAATGGGTCAATTCTTACAAGAAACAATTAACGAGCATCGTAGAACTTTTGATCCTAGCCATTTACGCGATCTTCTCGACACCTACCTTTTTGAAATTCAAAAGGCGAACGAAGAAGGCACCGGCCATCACCTTTTTGACGGACGCGATCACGATCGCCAAATGCAACAAATCATGGGTGATTTATTCTCAGCGGGGATGGAAACCATCAAAAACACTTTATTATGGTCCATCTTGTTTATGCTCCACTATCCAGAAGTGATGAAATCGATTCAAGAAGAGCTCGATCAAGTGATTGGACGCAAGCGTTTACCAAAACTCGAAGATTTGTCTTATCTGCCTGTGACCGAAGCGACAATCTGCGAAGTGATGAGGGTTTCGAGCATAATTCCCATGGGCACAACGCACGCACCCACACACGATATTCACTTGAACGGCTTCAGCGTACCTCACTACGCTCAGGTGGTGCCTCTTTTGCACGCAGTTCACATGGATCCAAATTTGTGGGACGAACCTGACAAATTCAACCCGTCTAGGTTTATCGATGGGGAAGGCAAAGTCAATAAGCCAGAATATTTCATACCCTTTGGTGTAGGACGGCGGATGTGTTTGGGAGAGATTTTAGCCCGAATGGAAGTGTTCCTGTTCTTCTCCACGTTACTTCACACGTTCGATCTTAGCGTGCCCGAAGGAGAAAAGTTACCTAGTTTAAAAGGCAATGCGGGCGTCACAATATTTCCAGATACGTTCAAAGTATGTGTCAAGCCAAGACCGTTAGAAGGCGAATACACATCATCGACGATACGATCAGCTGGCAGCCATTAA

>SPO_UNIGENE19886P26_RTSEQ_PARTIAL_1038BP

ATGCGCTTCCACAAGCTCTTCGGAGGAGACCGCAACAATTCACTTGCCCTCTGTGATTGGTCCTCATTGCAGAAGACACGACGAAGTATTGCTCGCATTTACTGCGCTCCAAAATTCACTTCTTTGCAGTACGACGATGTCAATAAAGTCGGTTGCGAAGCTGTCGAAGTGCTGCTTTCCGAATTGCAGAAGGAACCGATGGGACAACCGGTACAAGTAAAACCACTAATTTTGGCGGCGTGCGCAAATATGTTCACCCAGTACATGTGTTCAATAAATTTCGATTACCGAGACTTGGAGTTCCAAAAAATCGTCAGATACTTCGACGAAATATTTTGGGAAATCAATCAAGGTTACGCTGTCGATTTCCTGCCGTGGTTACTTCCGTTCTATTCGGGTCACATGAAGAAGATATCAGATTGGGCTAGCGAAATTAGGAAATTTATTTTGTCGCGGATTATTGATCAGCACAGAGCTTCTTTGGATCGTAACGTGCCGCCAAAAGATTTCACCGACGCCCTCCTGTTACACCTAGATGAAGACCCAAACCTGAATTGGCAACACATTATTTTCGAATTAGAAGATTTTCTAGGTGGGCATTCCGCCGTCGGCAATTTAGTAATGGTTACTCTCGCCGCTATAGTGAAATACCCGGAAATTGCTAAACGAATTCAAGCCGAAGTTGATTTAGTAACAGCTGGTACGAGATATCCAAATTTATTCGACAGAAGTGCCATGCCGTTTACGGAAGCTACCTTATGGGAAACTTTAAGAACGGCATCTTCACCTATTGTCCCTCATGTTGCAACGACAGACACGGAAATTGACGGCTATACCGTTAAAAAAGGCACGATGGTGTTTGTAAACAACTACGAGCTTAATGTTGGTGCCGATTATTGGAATCAACCGCACGAATTCAAACCGGAACGGTTTATTTCCGAAGACGGAACTGTAGTCAAGCCATCCCATTTCATTCCTTTCAGCACGGGAAAACGCACATGCATCGGCCAACGACTTGTTCAGTGCTTCAGCTTTGTA

>SPOT_UNIGENE12798_RTSEQ_PARTIAL_1011BP

GGACACCATTTCGATAGCAGACCCAACTTCGAACGCTACCAGCACCTATTCGGCGGCGACAAACAGAACTCTTTGGCCTTTTGCGACTGGTCGGAGAAACAGCGCGTAAGACGCGAGATGCTCAAGACGTACACGTTTCCGCGCGCAGCCTCGAACAAGTTCGCCTCACTCGAGGAGATCATCGAGATGGAGACGTCCAACATGGTGGCGCGCGTCGGCACCACCTGCATAAAAAAACTGATTCTGCACAGCTGCGCCAATATCTTCACGAACCACTTTTGCAGCAAGGATTTCGCCTTCGGCGACGAGCGTTTTGGTCGAATGATCGAAAACTTTGACGAAATCTTCTATGAAGTCAATCAGGGTTACGCCGCCGATTTTTTGCCGTTTCTTATGCCGTTGCATCAAGCTCGCTTGGAGAAGATGAGCGAGCTAGCTCACGAAATCCGTTCGTTTCTTGTGGAGAACGTTGTCGCAGGACGCTACGAAAATTATTCTGGGTACGAGCCCGCTGATTACGTCGAGAGTCTGATTAAGCACGTGAAAGACGAAGAAGGTTCGTACTTCAACTGGGAGTGCGCGTTGTTTGCTTTGGAAGACATCATAGGCGGGCACTCAGCTGTCGGAAACTTTTTAGTAAAGCTTCTCGGGTACCTGGTGCAAAACCCGCGCGTACAAGTCGAGATACAAAAAGAACTGGATGGGCTCGGTTTTAAAGCGTCGATTTCGGACCGCGCCTCGATGCCTTACACCGAAGCCACAATCCTAGAAGCTATCAGATTGATAGCGTCTCCGATCGTACCCAGAGCAGCCAACCAAGACACTTCCATCAACGGTTTCAGAATACCAAAAGGCACCCTCGTCTTCCTCAACAACTACGACTTAAGCATGTCGGAAAAACTATGGGACGAACCCGAACAATTCAAGCCTGAAAGATTTATAGTCAATAACCACATCATCAAGCCCGAGCACTTTTTACCTTTCGGCGGTGGCAGACGCAGCTGCATGGGT
